# The G_q_ signalling pathway inhibits brown and beige adipose tissue

**DOI:** 10.1038/ncomms10895

**Published:** 2016-03-09

**Authors:** Katarina Klepac, Ana Kilić, Thorsten Gnad, Loren M. Brown, Beate Herrmann, Andrea Wilderman, Aileen Balkow, Anja Glöde, Katharina Simon, Martin E. Lidell, Matthias J. Betz, Sven Enerbäck, Jürgen Wess, Marc Freichel, Matthias Blüher, Gabi König, Evi Kostenis, Paul A. Insel, Alexander Pfeifer

**Affiliations:** 1 Institute of Pharmacology and Toxicology, University Hospital Bonn, University of Bonn, 53127 Bonn, Germany; 2 Research Training Group 1873, University of Bonn, 53127 Bonn, Germany; 3 Department of Pharmacology, University of California, San Diego, California 92093, USA; 4 Institute of Pharmaceutical Biology, University of Bonn, D-53115 Bonn, Germany; 5 Department of Medical and Clinical Genetics, Institute of Biomedicine, The Sahlgrenska Academy, University of Gothenburg, Gothenburg SE-405 30, Sweden; 6 Molecular Signalling Section, Laboratory of Bioorganic Chemistry, National Institute of Diabetes and Digestive and Kidney Diseases, Bethesda, Maryland 20892, USA; 7 Institute of Pharmacology, University of Heidelberg, D-69120 Heidelberg, Germany; 8 Department of Medicine, University of Leipzig, 04103 Leipzig, Germany; 9 Department of Medicine, University of California, San Diego, California 92093, USA; 10 PharmaCenter, University of Bonn, 53127 Bonn, Germany

## Abstract

Brown adipose tissue (BAT) dissipates nutritional energy as heat via the uncoupling protein-1 (UCP1) and BAT activity correlates with leanness in human adults. Here we profile G protein-coupled receptors (GPCRs) in brown adipocytes to identify druggable regulators of BAT. Twenty-one per cent of the GPCRs link to the G_q_ family, and inhibition of G_q_ signalling enhances differentiation of human and murine brown adipocytes. In contrast, activation of G_q_ signalling abrogates brown adipogenesis. We further identify the endothelin/Ednra pathway as an autocrine activator of G_q_ signalling in brown adipocytes. Expression of a constitutively active G_q_ protein in mice reduces UCP1 expression in BAT, whole-body energy expenditure and the number of brown-like/beige cells in white adipose tissue (WAT). Furthermore, expression of G_q_ in human WAT inversely correlates with UCP1 expression. Thus, our data indicate that G_q_ signalling regulates brown/beige adipocytes and inhibition of G_q_ signalling may be a novel therapeutic approach to combat obesity.

## Abstract (web summary)

Brown and beige adipose tissues contribute to organismal energy expenditure by generating heat. Here, Klepac *et al.* survey G protein-coupled receptors in brown fat and show that G_q_-coupled receptors inhibit expression of thermogenic proteins in mice and in human adipocytes.

## Graphical abstract

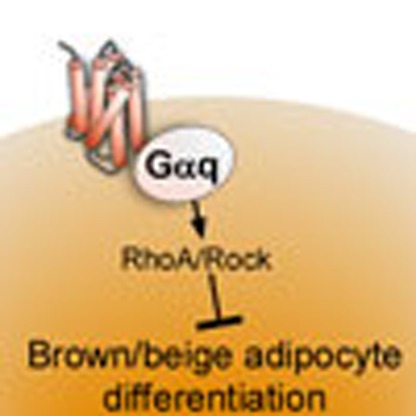

Brown adipose tissue (BAT) is important for basal and inducible energy expenditure in mammals^1^,^2^,^3^,^4^,^5^. Importantly, recent findings indicate that adult humans possess metabolically active BAT^6^ and that obese subjects have reduced activity of BAT^7^. BAT uniquely expresses the uncoupling protein-1 (UCP1), a mitochondrial protein which uncouples ATP production from oxidative phosphorylation leading to energy expenditure in a thermogenic manner. In contrast, white adipose tissue (WAT) is mainly responsible for energy storage and is an important endocrine tissue that releases adipokines, which in turn regulate energy intake and expenditure^8^. Brown-fat like cells have recently been described in WAT depots^9^ and their number and activity can be strongly induced by cold exposure, a process described as ‘browning'^2^,^10^.

G protein-coupled receptors (GPCRs) are a large family of seven transmembrane proteins^11^,^12^ that regulate important biological processes in diverse tissues including adipose tissue^13^,^14^. Approximately 25% of currently marketed drugs target GPCRs, illustrating their importance in disease and therapeutics. GPCRs are coupled to heterotrimeric G proteins, which are composed of Gα, β and γ subunits. Activation of GPCRs leads to the disassociation of Gα from the Gβγ dimer, allowing the binding and regulation of signalling effectors. The downstream signalling of GPCRs is in part determined by their G protein coupling^15^. There are four main classes of Gα proteins: G_s_, G_i_, G_q_ and G_12/13_. Activation of G_s_ and G_i_ leads to the stimulation or inhibition of cAMP signalling, respectively, while G_q_ activates phospholipase C (PLC). G_12/13_ activates the small GTPase Rho, a pathway also known to be modulated by the G_q_ family of proteins^16^,^17^.

Until now, the analysis of BAT GPCRs has focused mainly on a few G_s_-coupled receptors (for example, β-adrenergic^4^ and adenosine^18^ receptors) that activate cAMP signalling and UCP1-dependent thermogenesis. The function of other Gα GPCR families in adipocytes is not as clear. Therefore, in the present study, we investigate the expression pattern of non-odorant GPCRs in brown adipocytes (BAs). Our analysis reveals that G_q_-coupled receptors are highly expressed in brown adipocytes. Moreover, using pharmacological and genetic approaches, we found that G_q,_ via modulation of RhoA signalling, regulates adipogenesis of BAs *in vitro* and *in vivo*.

## Results

### GPCR expression of brown adipocytes

**Figure 1:**
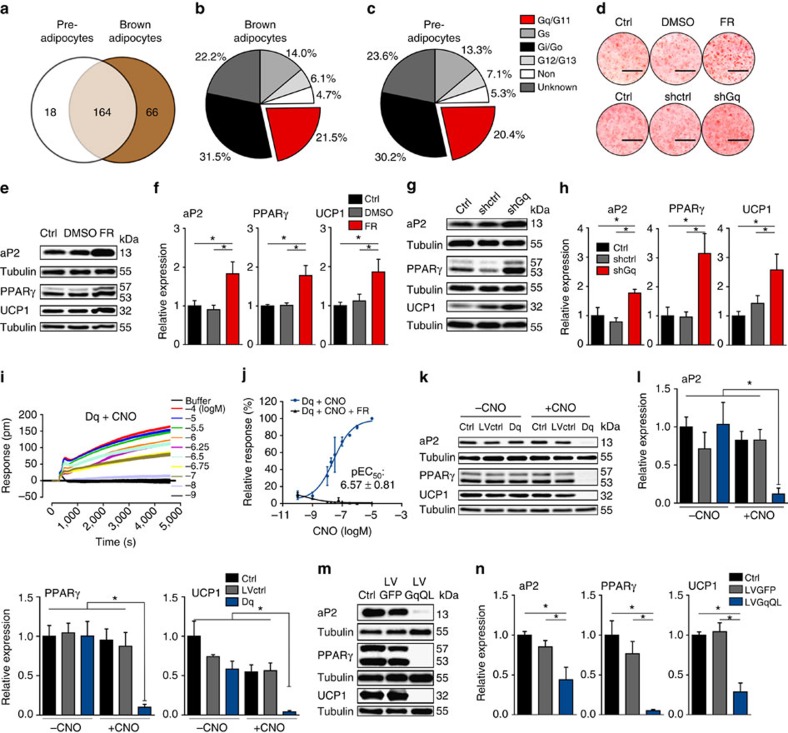
GPCR profiling and G_q_ signalling in murine brown adipocytes. (**a**) Venn diagram showing unique and overlapping GPCRs expressed in murine brown preadipocytes and mature murine BA. (**b**) Pie chart of GPCR-linkage classification in BA. (**c**) Pie chart of GPCR-linkage classification in brown preadipocytes. (**d**) Oil Red O stain of BA chronically treated with and without FR and after knockdown of G_q_ with shRNA (shGq); Ctrl, control. Scale bar, 1 cm. (**e**–**h**) Representative immunoblots (**e**,**g**) and quantification (**f**,**h**) of adipogenic markers (aP2; PPARγ) and thermogenic marker (UCP1) of BA after indicated treatment. ANOVA, *P<0.05. (**i**,**j**) Representative traces of label-free DMR (**i**) and concentration-effect curve (**j**) in cells stably expressing G_q_ DREADD (Dq) treated with indicated concentrations of CNO. (**k**,**l**) Representative immunoblots (**k**) and quantification (**l**) of aP2, PPARγ and UCP1 in BA transduced with control virus (LVctrl) or virus containing Dq and differentiated in presence or absence of CNO. ANOVA, *P<0.05. (**m**,**n**) Representative immunoblots (**m**) and quantification (**n**) of aP2, PPARγ and UCP1 in BA transduced with control virus (LVGFP) or virus expressing constitutively active G_q_ (LVGqQL). (**a**–**c**), *n*=3 independent arrays. (**d**–**h**) *n*=4. (**i,j**) *n*=3. (**k-l**) *n*=4. (**m**,**n**) *n*=5. ANOVA, *P<0.05. All data are shown as mean±s.e.m.

To identify the expression pattern of non-odorant/tastant GPCRs in BAs, we profiled the transcript levels of 347 GPCRs. We detected 182 GPCRs in preadipocytes and 230 in mature BAs (Supplementary Tables 1,2). 18 GPCRs and 66 GPCRs were uniquely expressed in preadipocytes and mature BAs, respectively (Fig. 1a). We used the IUPHAR database (http://www.guidetopharmacology.org/)^19^ to define GPCRs. Class A and adhesion GPCRs represented the two largest classes in both pre- and mature BAs (Table 1). During differentiation, class A receptors increased from 139 to 188 receptors, whereas 13 out of the 20 adhesion GPCRs expressed in preadipocytes decreased upon differentiation with five of them decreasing below the detection limit (Table 1). With respect to coupling to heterotrimeric G proteins, we found that 14.0 and 31.5% of the GPCRs detected in BAs interact with G_s_ and G_i_, respectively (Fig. 1b). Unexpectedly, 21.5% of the GPCRs expressed in BAs are predicted to link with G_q/11_ (Fig. 1b). Preadipocytes exhibited a similar pattern of GPCR linkage (Fig. 1c). During differentiation, the expression levels of 91 GPCRs changed more than twofold, with 26 and 65 showing a decrease or increase in expression, respectively (Supplementary Fig. 1a,b). During brown adipogenesis, G_q_- and G_i_-coupled GPCRs were the largest group of receptors with known G protein linkages to be down- and upregulated, respectively (Supplementary Fig. 1a,b).

To validate the expression pattern of GPCRs, we focused on six G_q_-coupled GPCRs that are highly expressed in pre- and/or mature BAs as identified by the GPCR array (Supplementary Tables 1,2): Endothelin (ET) receptors type A and B (*Ednra* and *Ednrb*), Adrenoceptor Alpha 1A (*Adra1a*), Angiotensin II receptor, type 1a and 1b (*Agtr1a* and *Agtr1b*) and Cholinergic Receptor, Muscarinic 3 (*Chrm3*). Both quantitative PCR (qPCR) and TaqMan GPCR array data demonstrated high expression of *Ednra* mRNA in preadipocytes and an increase of *Ednra* during differentiation (Supplementary Fig. 1c). *Ednrb* increased 280-fold (qPCR) and 332-fold (GPCR array) during differentiation; *Adra1a* increased 83-fold (qPCR) and 322-fold (GPCR array); *Agtr1a* increased 3.9-fold (qPCR) and 2.9-fold (GPCR array) (Supplementary Fig. 1c).

G_q_ and G_11_ are both expressed in preadipocytes, BAs and BAT and their levels of expression did not change significantly during differentiation (Supplementary Fig. 1d). Thus, GPCR/G_q/11_ signalling might contribute to the differentiation and function of BAs; however, virtually no information exists regarding this signalling pathway in BAT.

### Regulation of adipogenesis by G_q_

We inhibited G_q/11_ signal transduction using pharmacological and genetic approaches. Treatment with FR900359 (FR; 1 μM), a natural inhibitor of G_q/11_ (ref. 20), enhanced differentiation of murine BAs, as demonstrated by increased Oil Red O staining (Fig. 1d) and elevated protein levels of the adipogenic markers aP2 and PPARγ as well as of the thermogenic marker UCP1 (Fig. 1e,f). To complement the pharmacological approach, we used lentiviral small hairpin RNA (shRNA) directed only against G_q_ (shGq). Knockdown of G_q_ by ∼50% (Supplementary Fig. 1e) significantly increased adipogenesis, with increased lipid staining (Fig. 1d) and expression of adipogenic markers and UCP1 (Fig. 1g,h). Inactivation of G_q_ signalling thus promotes differentiation of murine BAs.

To determine the impact of increased G_q_ signalling on brown adipogenesis, we first selectively enhanced G_q_ signalling using a G_q_-coupled designer GPCR (DREADD)^21^ that represents a modified M3 muscarinic receptor (Dq)^22^. Dq couples exclusively to G_q_ and is activated by otherwise pharmacologically inactive clozapine-N-oxide (CNO)^22^. Brown preadipocytes were transduced with lentiviral vectors expressing Dq. To analyse G_q_-induced responses in living cells in a label-free manner, we used biosensor technology based on dynamic mass redistribution (DMR). Treatment of Dq-expressing cells with CNO (1 μM) induced a concentration-dependent DMR response with half-maximal activation at 269 nM (Fig. 1i,j). In contrast, no DMR response was observed in buffer- or CNO-treated wild-type cells (Supplementary Fig. 1f). The Dq-induced DMR response was markedly different from the optical traces obtained by stimulation of G_s_ with isoprenaline (Supplementary Fig. 1g,h), implying that DMR can be used to monitor different G protein signalling pathways in BAs. Treatment of Dq cells with FR abolished the CNO-induced DMR response, providing further evidence for FR as a G_q_ inhibitor (Fig. 1j and Supplementary Fig. 1i).

Dq stimulation during BA differentiation reduced Oil Red O staining (Supplementary Fig. 1j), suppressed expression of adipogenic markers aP2 and PPARγ and of UCP1 (Fig. 1k,l). Treatment of non-transduced control cells or cells transduced with a control virus (LVctrl) with CNO did not affect differentiation (Fig. 1k,l and Supplementary Fig. 1j). Expression of Dq without CNO stimulation did not alter BA differentiation (Fig. 1k,l and Supplementary Fig. 1j).

Secondly, we used lentiviral vectors to express a constitutively active mutant of G_q_ (LVGqQL) as a means to enhance G_q_ signalling. We validated the activity of GqQL in brown preadipocytes by measuring IP1 in non-transduced cells and cells that express either GFP or GqQL. IP1 is a downstream metabolite of PLC and IP3, a pathway known to be activated by G_q_ GPCRs. We found that IP1 levels were highly increased in preadipocytes that express GqQL compared with GFP-expressing or control cells (Supplementary Fig. 1k). GqQL overexpression during adipocyte differentiation reduced Oil Red O staining and inhibited the expression of adipogenic and thermogenic markers (Fig. 1m,n and Supplementary Fig. 1l) in a similar manner as did the DREADD approach.

### Endothelin/Ednra regulation of brown adipocytes

**Figure 2:**
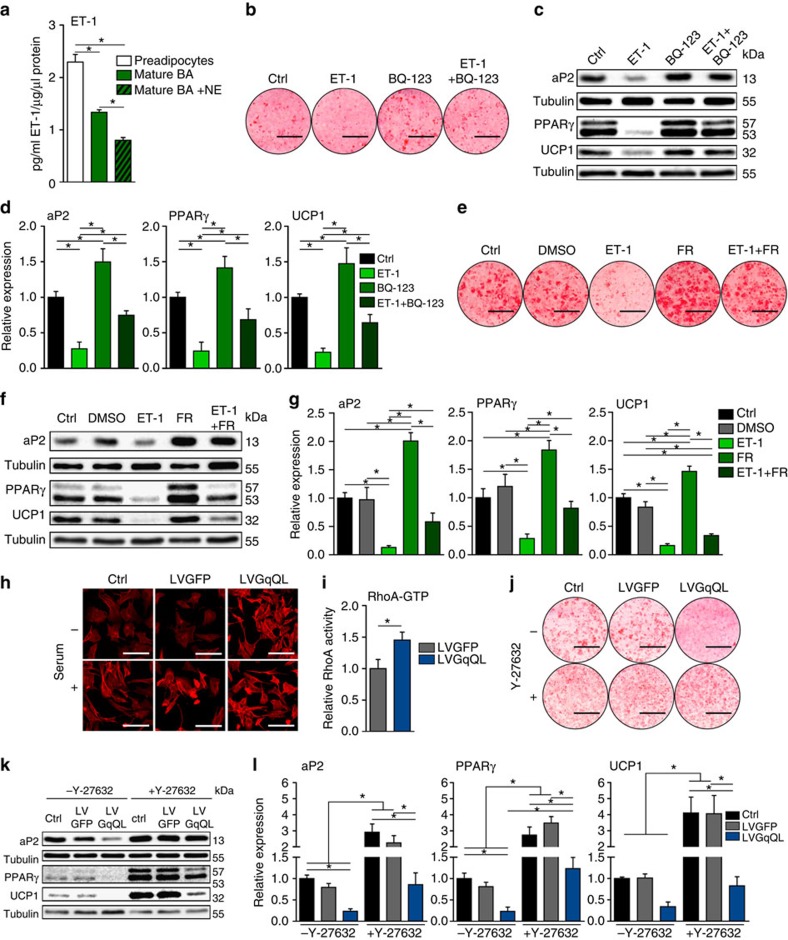
Role of Endothelin-1 in differentiation and downstream signalling of G_q_. (**a**) Release of ET-1 by preadipocytes, non-stimulated and NE-stimulated BA. ANOVA, *P<0.05. (**b**) Oil Red O stain of BA chronically treated with ET-1, Ednra inhibitor BQ-123, or both. Scale bar, 1 cm. (**c**,**d**) Representative immunoblots (**c**) and quantification (**d**) of adipogenic markers and UCP1 after treatment of BA with and without ET-1, BQ-123 or both. ANOVA, *P<0.05. (**e**) Oil Red O stain of BA chronically treated with ET-1, FR, or both. Scale bar, 1 cm. (**f**,**g**) Representative immunoblots (**f**) and quantification (**g**) of aP2, PPARγ and UCP1 of BA chronically treated with and without ET-1, FR or both. ANOVA, *P<0.05. (**h**) Phalloidin staining of F-Actin stress fibres in non-transduced cells and cells transduced with control virus (LVGFP) or virus expressing constitutively active G_q_ (LVGqQL) in serum-starved and stimulated state. Scale bar, 50 μm. (**i**) RhoA activation assay in preadipocytes expressing GFP (LVGFP) or GqQL (LVGqQL). *t*-test, *P<0.05. (**j**) Oil Red O stain of non-transduced BA and BA expressing GFP (LVGFP) or GqQL (LVGqQL) differentiated in presence or absence of ROCK inhibitor Y-27632. Scale bar, 1 cm. (**k**,**l**) Representative immunoblots (**k**) and quantification (**l**) of aP2, PPARγ and UCP1 in BA non-transduced and transduced with lentivirus expressing GFP (LVGFP) or GqQL (LVGqQL) and differentiated in presence or absence of Y-27632. (**a**–**d**) *n*=4. (**e**–**i**) *n*=3. (**j**–**l**) *n*=5. ANOVA, *P<0.05. All data are shown as mean±s.e.m.

To identify an endogenous receptor that activates G_q_ signalling in BAs, we focused on Ednra, one of the most highly expressed G_q_-coupled GPCRs in preadipocytes and BAs (Supplementary Tables 1,2). Both preadipocytes and BAs express constituents of the ET system: Endothelin-1 (*ET-1*), ET receptors type A and B (*Ednra* and *Ednrb*) and ET converting enzyme-1 (*ECE-1*) (Supplementary Fig. 2a). Although *ECE-1* was expressed at low levels in BA, it is not the only enzyme that can catalyse the production of ET-1 (ref. 23). Importantly, ET-1 was released by both cell types (Fig. 2a) and its secretion was inhibited by noradrenaline (NE) (Fig. 2a).

Differentiation of BAs was inhibited by chronic treatment with ET-1 (0.3 nM) but was restored by the selective Ednra antagonist BQ-123 (100 nM) (Fig. 2b–d) and by treatment with FR (1 μM) (Fig. 2e–g). Notably, BQ-123 increased the differentiation of the untreated cells. In contrast, ET-1 effects on differentiation were not restored with the Ednrb antagonist BQ-788 (100 nM) (Supplementary Fig. 2b–d). Moreover, acute treatment of mature BAs with ET-1 reduces lipoprotein lipase gene expression^24^. Lipoprotein lipase is important for fatty acid uptake and fuels cold-/NE-induced BAT thermogenesis^25^. Together these data implicate ET-1 as an autocrine/paracrine inhibitor of BA function that signals via Ednra/G_q_ and is regulated by NE signalling.

### G_q_ regulation of BAs via Rho/Rho-kinase

G_q_ can activate multiple intracellular signalling pathways including IP3/DAG, Ca^2+^, ERK and also by GTP-binding proteins of the Ras and Rho families^16^,^17^. Since Rho/Rho-kinase (ROCK) controls brown adipogenesis^26^,^27^, we evaluated this pathway. Cells expressing GqQL had a marked increase in phalloidin staining of actin filaments (Fig. 2h) and Rho activity (Rho-GTP pull-down assay) (Fig. 2i). In addition, pharmacological inhibition of ROCK with Y-27632 (10 μM) enhanced differentiation and increased expression of adipogenic and thermogenic markers in GqQL-expressing BAs (Fig. 2j–l). ROCK inhibition also restored adipogenesis of ET-1 treated cells (Supplementary Fig. 2e–g). These results suggest that G_q_ inhibits BA differentiation via the RhoA/ROCK pathway.

### *In vivo* regulation of BAT by G_q_ signalling

**Figure 3:**
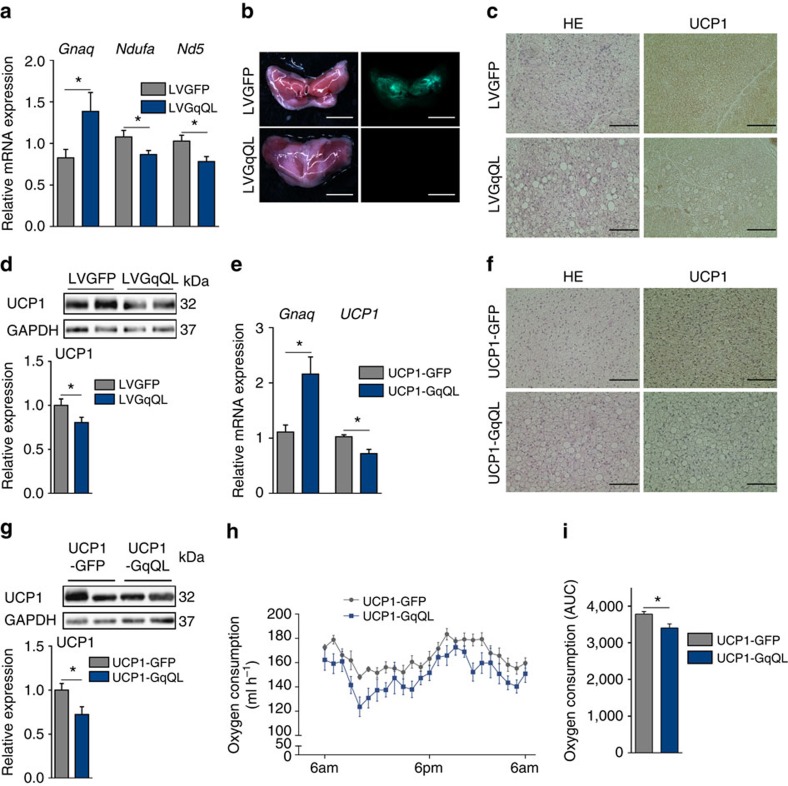
G_q_ activation negatively affects BAT *in vivo*. (**a**) Relative mRNA expression of *G*_*q*_, *Ndufa* and *Nd5* in BAT of LVGFP and LVGqQL mice. *t*-test, *P<0.05. (**b**) Representative images of interscapular BAT of mice injected with LVGFP or LVGqQL in bright field (left) and fluorescent microscopy (right). Scale bar, 5 mm. (**c**) Representative hematoxylin/eosin (HE) and UCP1 staining of BAT in LVGqQL and LVGFP mice after cold stimulation. Scale bar, 100 μm. (**d**) Representative immunoblots (top) and quantification (bottom) of UCP1 expression in BAT of LVGFP and LVGqQL mice. *t*-test, *P<0.05. (**e**) Relative mRNA expression of *G*_*q*_ and *UCP1* in BAT of UCP1-GFP and UCP1-GqQL transgenic mice. *t*-test, *P<0.05. (**f**) Representative HE and UCP1 staining of BAT in UCP1-GFP and UCP1-GqQL mice after cold stimulation. Scale bar, 100 μm. (**g**) Representative immunoblots (top) and quantification (bottom) of UCP1 expression in BAT of UCP1-GFP and UCP1-GqQL mice. *t*-test, *P<0.05. (**h**) Oxygen consumption of UCP1-GFP and UCP1-GqQL mice over 24 h. (**i**) Area under the curve (AUC) of the oxygen consumption of UCP1-GFP and UCP1-GqQL mice over 24 h *t*-test, *P<0.05. (**a**–**d**) 11 animals per group were analysed. (**e**,**g**) 6 animals per group were analysed., (**f**,**h**,**i**) 4 animals per group were analysed. All data are shown as mean±s.e.m.

To study the effects of elevated G_q_ signalling *in vivo*, we expressed constitutively active G_q_ in BAT (Fig. 3a). Lentiviral particles encoding either GqQL (LVGqQL) or GFP (LVGFP) under the control of the CMV promoter were directly injected into BAT of mice that were subsequently cold-exposed for 2 weeks (Fig. 3b). Histological analysis revealed increased size of the lipid droplets with more unilocular, white-like adipocytes in LVGqQL-injected BAT and decreased UCP1 staining (Fig. 3c). Expression of GqQL decreased UCP1 protein level in BAT by ∼20% (Fig. 3d) and decreased expression of the mitochondrial marker genes *Nd5* and *Ndufa* (Fig. 3a).

In addition, we generated transgenic mice that express either GqQL or GFP (control) under the control of the UCP1 promoter (UCP1-GqQL and UCP1-GFP, respectively) to achieve BA-specific expression (Fig. 3e), and exposed them to cold. Similar to what occurred after injection of LVGqQL, immunohistochemical staining of BAT from UCP1-GqQL mice revealed enlarged, unilocular lipid droplets and reduced UCP1 staining (Fig. 3f). UCP1 expression of BAT of UCP1-GqQL mice was significantly decreased compared with the control UCP1-GFP group (Fig. 3e,g). Indirect calorimetry revealed significantly reduced O_2_ consumption in cold-exposed UCP1-GqQL mice (Fig. 3h,i). Together, these results indicate that G_q_ activation induces a phenotypic change of BAT towards WAT (a process also known as ‘whitening'^28^) with diminished thermogenic capacity of BAT.

**Figure 4:**
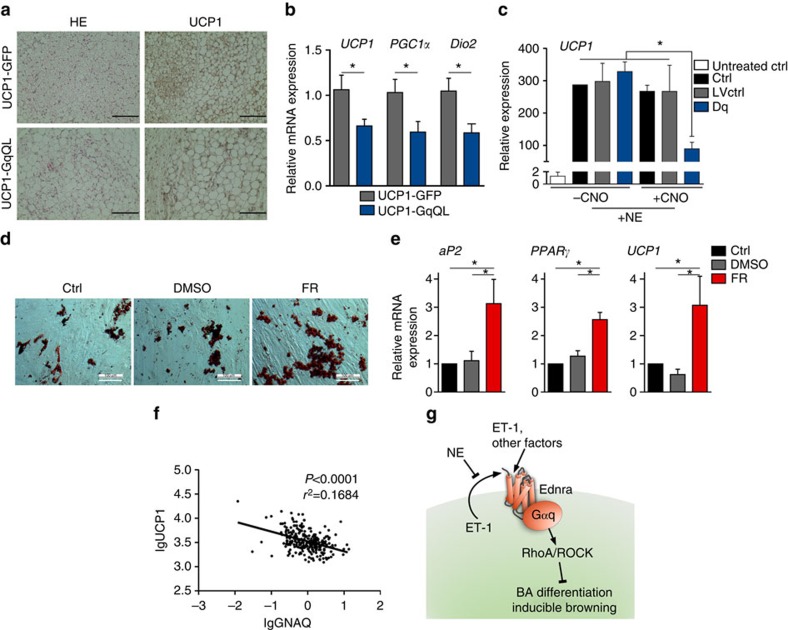
G_q_ activation diminishes browning of WAT *in vivo*. (**a**) Representative HE and UCP1 staining of inguinal WAT in UCP1-GFP and UCP1-GqQL mice after cold stimulation. Scale bar, 100 μm. (**b**) Relative mRNA expression of thermogenic markers in inguinal WAT of UCP1-GqQL relative to UCP1-GFP mice. *t*-test, *P<0.05. (**c**) *UCP1* expression in NE-stimulated murine brown/beige-like adipocytes transduced with control virus (LVctrl) or lentivirus carrying G_q_ DREADD (Dq) in presence and absence of CNO. ANOVA, *P<0.05. (**d**) Oil Red O stain of human brown/beige adipocytes (hMADS) differentiated in the presence or absence of FR. Scale bar, 100 μm. (**e**) Relative mRNA expression of adipogenic markers (*aP2*; *PPAR*γ) and *UCP1* of hMADS differentiated in the presence or absence of FR normalized to *GAPDH* (Glyceraldehyde 3-phosphate dehydrogenase). ANOVA, *P<0.05. (**f**) Correlation of *G*_*q*_ and *UCP1* expression in human WAT. (**g**) Scheme of ET-1/G_q_ signalling in BA differentiation and browning. (**a**,**b**) 6 animals per group were analysed. (**c**–**e**) *n*=3. (**f**) 266 human abdominal WAT samples were analysed. All data are shown as mean±s.e.m.

Apart from classical interscapular BAT, inducible brown (also known as beige/brite) adipocytes have been identified in murine and human WAT^2^,^10^. Exposure of transgenic UCP1-GqQL mice to cold, induced a 1.9-fold and 1.5-fold increase in UCP1 promoter-driven G_q_ expression in inguinal (iWAT) and gonadal/visceral (gWAT) WAT, respectively (Supplementary Fig. 3a,b). Histological analysis of both iWAT and gWAT revealed ‘browning' in the control mice that express GFP under the control of the UCP1 promoter (Fig. 4a and Supplementary Fig. 3c). In contrast, iWAT and gWAT of GqQL mice exhibited larger lipid droplets with decreased expression of BA markers (*UCP1*, *PGC1-α* and *DIO2*) (Fig. 4a,b and Supplementary Fig. 3c,d).

Further evidence for a role of G_q_ in inducible brown/beige cells was obtained by induction of browning in isolated primary murine adipocytes (Fig. 4c). Using the DREADD system (Fig. 1i–l), we activated G_q_ signalling with CNO (10 μM), which resulted in a significant reduction in *UCP1* expression (Fig. 4c).

To explore the effect of enhanced G_q_ signalling on white adipogenesis, we used murine white adipocytes isolated from iWAT (WAi). We overexpressed Dq and activated it with CNO throughout differentiation. G_q_ activation suppressed WAi differentiation (Supplementary Fig. 3e–g).

### Role of G_q_ signalling in human adipocytes

Human BAT also highly expresses G_q_ protein. We found G_q_-coupled receptors that were highly expressed in murine BAs are also highly expressed in the human brown/beige adipocyte cell line (hMADS) (*Ednra*, *Ednrb, Adra1a, Agtr1* and *Chrm3*) (Supplementary Fig. 3h,i). Treatment of the hMADS cells with FR (1 μM) enhanced differentiation and expression of adipogenic markers *aP2* and *PPAR*γ and thermogenic marker *UCP1* (Fig. 4d,e). Moreover, we found a relationship between expression of G_q_ and the thermogenic markers *UCP1* and *CIDEA* in WAT samples from 266 human subjects (Fig. 4f and Supplementary Fig. 3j). *UCP1* and *CIDEA* expression were both significantly inversely correlated with G_q_; results that are consistent with the idea that G_q_ expression negatively affects the thermogenic capacity in humans (Fig. 4f and Supplementary Fig. 3j).

## Discussion

In the present study, we screened for non-chemosensory GPCRs in murine brown preadipocytes and mature adipocytes. GPCR profiling revealed that BAs express more than 180 GPCRs and that the expression pattern changes greatly upon differentiation. The number of GPCRs detected is in agreement with previous analysis performed on human subcutaneous adipose tissue and murine metabolic tissues that detected 163 and 198 GCPRs, respectively^29^,^30^. In BAs, we found that G_q_-coupled receptors were one of the largest groups and that their expression was highly altered during differentiation, indicating a potential role in adipogenesis. Importantly, we show that G_q_ signalling is a crucial inhibitor of adipocyte differentiation, through activation of the Rho/ROCK signalling cascade, a major regulator of BA differentiation^26^ (Fig. 4g).

Treatment with the G_q_ inhibitor FR enhanced the adipogenic and thermogenic potential of BAs, indicating either the presence of constitutively active G_q_ receptors and/or an autocrine loop of G_q_ signalling in BAs. Analysis of highly expressed G_q_-coupled GPCRs indicated that murine BAs and human brown/beige cells express ET receptors. Moreover, murine BAs also express the enzymes necessary for ET production and secretion. Stimulation with ET-inhibited adipogenesis, while the inhibition of G_q_ and Ednra, but not Ednrb, rescued adipogenesis of ET-treated cells. Recently, Xue *et al.*^31^ identified Endrb as a genetic marker that positively correlates with the thermogenic capacity of cloned human BAs. Inhibition of Ednra alone increased the differentiation of untreated cells, indicating endogenous activity of the ET system in BAs. Thus, our data implicate the ET-1/Ednra signalling axis as an autocrine/paracrine inhibitor of brown/beige adipogenesis via G_q_ signalling (Fig. 4g). In addition to its function in BAT, ET-1 plays an important role in the vasculature and excessive ET-1 contributes to atherosclerosis^32^ as well as to the disturbed insulin-induced vasorelaxation in obesity^33^.

Our data also indicate an inhibitory role of G_q_ in BAT function and browning *in vivo*. Overexpression of GqQL induced a phenotype of BAT reminiscent of ‘whitening', while preventing the ‘browning' of WAT in mice exposed to cold. Overall, activation of G_q_ signalling in brown/beige adipocytes resulted in reduced whole-body energy expenditure of cold-exposed mice. Thus, our data show that the G_q_ signalling pathway inhibits brown and beige adipose tissue *in vivo* and the studies done using cultured adipocytes show that *G*_*q*_ signalling inhibits adipogenesis in a more general manner. Furthermore, we found a negative correlation between G_q_ and *UCP1* mRNA expression in human abdominal WAT. Although abdominal adipose tissue is predominantly white, it can be induced to brown and inducible brown-like cells have been identified in abdominal fat after β-3 adrenergic receptor treatment^34^. Further studies are required to investigate the role of alterations in the expression of BAT marker genes in abdominal fat depots in adult humans. Interestingly, G_q_ signalling is not only involved in regulation of adipocyte differentiation and non-shivering thermogenesis, but also in the CNS regulation of appetite^35^.

In conclusion, our results demonstrate a previously unappreciated role in brown/beige adipogenesis for G_q_, to which ∼20% of the GPCRs expressed in these cells are predicted to couple. Our findings suggest that antagonizing G_q_ signalling by targeting G_q_-coupled GPCRs and/or by directly inhibiting G_q_ represent previously unexplored ways to enhance the amount of brown/beige fat and thus increase energy expenditure.

## Methods

### Isolation and differentiation of BAs

BAT-derived mesenchymal stem cells were isolated from interscapular BAT of newborn wild-type mice^26^,^36^. Excised BAT was incubated for 30 min at 37 °C in digestion buffer (Dulbecco's modified Eagle's medium (DMEM, Invitrogen) containing 123 mM Na^+^, 5 mM K^+^, 1.3 mM Ca^2+^, 131 mM Cl^−^, 5 mM glucose, 1.5% (w/v) bovine serum albumin (BSA), 100 mM Hepes and 0.2% (w/v) collagenase type II (pH 7.4)). After digestion, tissue remnants were removed by filtration through a 100 μm nylon mesh and placed on ice for the next 30 min. The infranatant was filtered through a 30 μm nylon mesh and centrifuged at 700*g* for 10 min. The pellet containing mesenchymal stem cells was resuspended in DMEM supplemented with 10% fetal bovine serum (FBS), 100 IU penicillin, streptomycin (100 μg ml^−1^) (P/S), 4 nM insulin, 4 nM triiodothyronine, 10 mM Hepes and sodium ascorbate (25 μg ml^−1^). Approximately 60,000 cells per cm^2^ were seeded on a six-well plate and grown at 37 °C and 5% CO_2_. Preadipocytes were immortalized using the lentivirus containing the SV40 large T antigen and unselected cells were cultured in DMEM supplemented with FBS and P/S (GM). The cells were expanded in GM at 37 °C, 5% CO_2_ (ref. 37).

For differentiation, immortalized cells were seeded in GM on a six-well plate in a density of ∼180,000 cells per well. 48 h after seeding (day −2), GM was replaced by differentiation medium (DM), supplemented with FBS, P/S, 20 nM insulin and 1 nM triiodothyronine. Confluent cells (day 0) were then treated for 48 h with BAT induction medium, which was DM supplemented with 0.5 mM isobutylmethylxanthine (IBMX) and 1 μM dexamethasone. After the induction, cells were treated with DM for the next 5 days, which was replenished every second day. Where indicated, cells were treated every second day with the G_q_ inhibitor FR (1 μM), ROCK inhibitor Y-27632 (Tocris, 10 μM), CNO (Tocris, 1 μM), Endothelin-1 (ET-1) (Tocris, 0.3 nM), Ednra inhibitor BQ-123 or Ednrb inhibitor BQ-788 (both Tocris, 100 nM). In the experiments with ET-1; FR, Y-27632, BQ-123 or BQ-788 were added to the culture medium 30 min before the addition of ET-1.

For all the preadipocyte experiments, the cells were used on day −2. For mature BA experiments, the cells were used on day +7 after induction.

### Isolation and differentiation of white and beige adipocytes

WAT-derived mesenchymal stem cells were isolated from inguinal white fat pads of 8- to 12-week-old wild-type mice. Excised WAT was incubated for 30 min at 37 °C in digestion buffer (DMEM containing 0.5% (w/v) BSA and 0.15% (w/v) collagenase type II). After digestion, cells were allowed to stand for 10 min at room temperature and centrifuged at 700*g* for 10 min. The pellet was resuspended in GM and filtered through a 100 μm nylon mesh. Approximately 180,000 cells per well were seeded in GM on a six-well plate at 37 °C and 5% CO_2_.

For differentiation, cells were induced 2 days after reaching confluence (day 0) for 48 h, using WAT induction medium (DMEM supplemented with 5% FBS, 1% P/S, 1 μM dexamethasone, 0.5 mM IBMX, 1 nM triiodothyronine, 1 mM D-biotin, 17 mM pantothenate, L-ascorbate (50 mg ml^−1^), 1 μM rosiglitazone and 0.172 mM insulin). After the induction, cells were treated with maintainance medium (DMEM supplemented with 5% FBS, P/S, 1 nM triiodothyronine, 1 mM D-biotin, 17 mM pantothenate, L-ascorbate (50 mg ml^−1^), 1 μM and 0.172 μM insulin) for the next 7 days, which was replenished every second day. For differentiation experiments, cells were treated every second day with CNO (1 μM). Browning was induced by 8 h NE (1 μM) stimulation, in the presence or absence of CNO (10 μM).

For all the preadipocyte experiments, the cells were used on day −2. For mature white adipocyte experiments, the cells were used on day +7 after induction.

### hMADS differentiation

Human multipotent adipose-derived stem cells were provided by the laboratory of C. Dani (University of Nice SophiaAntipolis)^38^ and differentiated into brown/beige cells as follows^18^,^39^. Cells were seeded on 12-well plates in growth medium (DMEM Low Glucose (Lonza), supplemented with 1x glutamine (Lonza), 10 mM Hepes buffer (Lonza), penicillin-streptomycin 5,000 IU ml^−1^ to 5,000 UG ml^−1^ (Lonza) and 10% FBS (S.A Dutscher, Brumath, France)) containing 2.5 ng ml^−1^ FGF2 (Peprotech) at a density of ∼160,000 cells per well. After 48 h, medium was replaced with growth medium containing no FGF2. When the cells reached confluency, growth medium was replaced by hMADS induction medium (day 0) (growth medium supplemented with 5 μg ml^−1^ insulin, 10 μg ml^−1^ transferrin, 0.2 nM triiodothyronine, 1 μM rosiglitazone, 100 μM IBMX and 1 μM dexamethasone) for the next 72 h. Cells were then cultured in differentiation medium (induction medium without IBMX and dexamethasone) for 9 more days. Where indicated, cells were treated from day 0 to day 12 with the G_q_ inhibitor FR (1 μM).

For all the pre-adipocyte experiments, the cells were used on day −2. For mature BA experiments, the cells were used on day +12 after induction.

### RNA isolation and real-time RT-PCR (qPCR)

RNA was isolated using Trizol method (Analytik Jena AG). The samples from human retroperitoneal BAT have been previously described^40^. cDNA was synthesized from 0.5 μg RNA using Transcriptor First Strand cDNA Synthesis Kit (Roche). Real-time RT-PCR (qPCR) was performed with SYBR-Green PCR master mix (Applied Biosystems) or LightCycler 480 SYBR Green I Master (Roche) using a HT7900 instrument (Applied Biosystems). Fold changes were calculated using relative quantification methods with mHPRT (murine hypoxanthine guanine phosphoribosyl transferase) serving as an internal control unless otherwise stated. Primer sequences are available in Supplementary Table 3.

### GPCR expression analysis

GPCR profiling was performed using TaqMan Mouse GPCR Arrays (Applied Biosystems) for quantitative expression analysis of mouse GPCR genes^41^. These arrays detect 347 non-chemosensory GPCRs. cDNA was synthesized from 0.5 μg RNA using Transcriptor First Strand cDNA Synthesis Kit (Roche). Two nanograms of cDNA was used for each gene in the GPCR arrays. Quantification of GPCR cDNA expression was normalized to 18S rRNA. Data shown are an average of three independent arrays. GPCRs were considered unexpressed if at least two of the arrays had a delta *C*_t_ above 25. G protein coupling of GPCRs was determined using the IUPHAR database (http://guidetopharmacology.org/).

### Lentiviral infection

Constitutively active *G*_q_ (GqQL), was provided by Silvio Gutkind, NIH^42^ Constitutive activity was achieved by mutating Glutamine to Leucine at the amino acid residue 209. Lentiviral vectors were obtained by cloning constitutively active G_q_ into the Bam HI and Sal I sites of the vector p156rrlsinPPTCMV, which carries a murine uncoupling protein-1 promoter (UCP1-GqQL) or ubiquitous CMV promoter (LVGqQL). Control vectors (p156rrlsinPPT) contain green fluorescent protein (UCP1-GFP and LVGFP).

The shRNA against G_q_ and the control shRNA were purchased from Sigma Aldrich and were expressed under the U6 promoter (pLKO.1-U6-sh- ctrl sequence: 5′-GCATGCAGAAGTGTAAAGCTA-3′, pLKO.1-U6-sh-Gq-287918-PGK-Puro 5′-GCTTGTGGAATGATCCTGGAA-3′).

pLenti6.3V5-HA-hM3D(Gq)-mCherry (Dq) lentiviral vectors were obtained from Bryan Roth, University of North Carolina. pLenti6.3-CMV-TO-GFP-Blasticidin was used as the control virus (LVctrl) for the Dq experiments.

Lentiviral particles were generated by transfection of HEK293T cells with vector constructs and packaging plasmids^26^,^36^. Viral particles were concentrated by ultracentrifugation^26^,^36^. For the lentiviral infection, cells were seeded on a six-well plate and transduced with lentiviruses corresponding to 50 ng (RTase quantified by ELISA) UCP1-GqQL, UCP1-GFP, LVGqQL or LVGFP; 75 ng Dq or LVctrl and 100 ng of the shRNA viruses for 12–16 h. Cells were further differentiated as described above.

### Oil Red O staining

Mature BAs were fixed in PBS containing 4% paraformaldehyde (PFA). After washing with PBS, cells were incubated with Oil Red O (Sigma) solution (3 mg ml^−1^ in 60% isopropyl alcohol) for 3 h at room temperature, washed with PBS and visualized under a microscope.

### Western blot analysis

Protein lysates from cells and tissues were isolated^26^,^36^ using lysis buffer (50 mM Tris, pH 7.5, 150 mM sodium chloride, 1% NP-40, 0.5% sodium deoxycholate, 0.1% SDS, 0.1 mM EDTA and 0.1 mM EGTA) supplemented with complete protease inhibitor cocktail (Roche), 1 mM Na_3_VO_4_ and 10 mM NaF. Protein contents were determined by the Bradford method. Proteins were separated using SDS–polyacrylamide gel electrophoresis and transferred onto a nitrocellulose membrane.. Membrane was blocked for 1 h in 5% BSA in Tris-Buffered Saline and 0.1% Tween 20 (TBST) and incubated over night at 4 °C in different primary antibodies (all primary antibodies diluted 1:1,000). Incubation in secondary antibody was performed the next day, for 1 h at room temperature in 5% milk in TBST. Proteins were visualized with an enhanced chemiluminescence (ECL) reagent and quantified by densitometric analysis with Image J software. Antibodies directed at the following were used: aP2 (Santa Cruz Biotechnology, Cat#: sc-18661), PPARγ (Santa Cruz Biotechnology, Cat#: sc-7273); UCP1 (Sigma Aldrich, Cat#: sc-6529 and Thermo Fisher Scientific, Cat#: PA1-24894); Tubulin (Dianova, Cat#: MS-719-P0) and GAPDH (Cell Signalling, Cat#: 2118). Secondary horse radish peroxidase–linked antibodies against goat (Dianova, Cat#: 705-035-147, dilution 1:5,000), mouse (Dianova, Cat#: 115-035-146, dilution 1:10,000) and rabbit (Cell Signalling, Cat#: 7074, dilution 1:5,000) were used. Complete immunoblots of western blot sections are shown in Supplementary Fig. 4.

### Label-free dynamic mass redistribution assays

The DMR analysis was carried out using the Corning Epic system (Corning, NY, USA) in conjunction with a Cybi-SELMA semi-automated electronic pipetting system (Analytik Jena AG, Jena, Germany)^43^. Briefly, brown preadipoyctes stably expressing Dq were seeded on fibronectin-coated 384-well biosensor plates at a density of 3,000 cells per well and cultivated for 48 h in growth medium (37 °C, 5% CO_2_). Before the measurement, cells were washed twice with assay buffer (Hanks balanced salt solution with 20 mM HEPES). Where indicated, cells were pre-incubated with FR (1 μM) for 1 h or cholera toxin (CTX; 100 ng ml^−1^) for 18–20 h before measurement. DMR was monitored for at least 5,000 s. The optical DMR recordings were buffer-corrected. To quantify the DMR signals for concentration-effect curves, the maximum response within 1,000 s was calculated. pEC_50_ value determination and data calculation were performed using GraphPad Prism 5.04 (GraphPad Software, La Jolla, USA).

### IP1 assay

Intracellular alteration of the second messenger IP1 was quantified with the HTRF-IP1 kit (Cisbio Bioassays) following the manufacturer's instructions. Briefly, brown preadipocytes non-transduced and transduced with lentiviruses carrying GqQL (LVGqQL) or GFP (LVGFP) were cultivated for 48 h in growth medium (37 °C, 5% CO_2_). For the assay, cells were resuspended in stimulation buffer containing 50 mM LiCl and transferred to a 384-well microtiter plate at a density of 12,500 cells per well. After 90 min incubation time, 3 μl of IP1-d2 conjugate followed by 3 μl of europium cryptate-labelled anti-IP1antibody solved in lysis buffer were added to the cells to quantify intracellular IP levels. After a further incubation of 1 h at room temperature, time-resolved fluorescence was measured at 620 and 665 nm with the Mithras LB 940 multimode reader.

### ET-1 ELISA assay

Cell culture medium was collected from the preadipocytes and mature BAs, with or without 8 h NE (1 μM) stimulation. ET-1 ELISA was performed using the Endothelin-1 ELISA kit (Enzo) according to the manufactureŕs instructions.

### F-actin staining of adherent cells in culture

Glass coverslips were placed in 24-well plates and coated with fibronectin (5 μg ml^−1^). Brown preadipocytes were seeded at a density of 20,000 cells per well and serum-starved for 24 h. Cells were incubated for 30 min with 10% FBS to induce the formation of F-actin stress fibres. Cells were then fixed with 4% PFA, permeabilized with 0.1% Triton-100, blocked with 1% BSA/PBS and stained with phalloidin-Alexa 546. Coverslips were mounted on glass slides using PermaFluor mounting medium and visualized using a confocal microscope.

### RhoA activation assay

Brown preadipocytes were seeded on a 10 cm tissue plate at a density of 180,000 cells per plate and infected with lentiviruses carrying GqQL or GFP (control) for 8 h. Cells were cultured until reaching 50–60% confluency, serum-starved for 24 h and collected for RhoA activity measurement. RhoA activation was assessed with an ELISA-based RhoA G-LISA Activation Assay Kit (Cytoskeleton). The level of RhoA activity was determined by colorimetric measurement at 490 nm. Active RhoA was normalized to total RhoA protein.

### Lentiviral injections of constitutively active G_q_ (CMV-GqQL) into BAT

Four-week-old male mice (C57BL/6, Charles River) were anaesthetized using isoflurane. A small incision was made in the neck region and 1 μg of lentiviruses carrying either GqQL (LVGqQL) or GFP (LVGFP) under control of the ubiquitous CMV promoter were injected directly into each fat pad of BAT. After injections, mice were acclimatized to cold for 1 week at 18 °C following 1 week of cold exposure at 4 °C. During the study, the mice were maintained on a daily cycle of 12 h light (0600 to 1800 hours) and 12 h darkness (1800 to 0600 hours), and were allowed free access to standard chow and water. The study was approved by the Landesamt für Natur, Umwelt und Verbraucherschutz, NRW, Germany.

### Generation of transgenic mice and energy expenditure

Transgenic mice were generated by subzonal injections^44^ of the lentiviruses UCP1-GqQL and UCP1-GFP into oocyte of a wild-type donor mouse with C57BL/6 background. Ten-week-old female mice were kept for one week at 18 °C followed by one week cold exposure at 4 °C. Oxygen consumption was measured with Phenomaster (TSE Systems) for 120 s every 16 min for 24 h. During the study, the mice were maintained on a daily cycle of 12 h light (0600–1800 hours) and 12 h darkness (1800–0600 hours), and were allowed free access to standard chow and water. The study was approved by the Landesamt für Natur, Umwelt und Verbraucherschutz, NRW, Germany.

### Immunohistochemistry

BAT and WAT was fixed in PBS containing 4% PFA for 48 h and dehydrated using ethanol. Tissue was embedded in paraffin and cut into 5 μM sections. Sections were blocked with 2% normal goat serum-TBS (tris-buffered saline) for 30 min RT and immunohistochemical stainings were performed with a primary antibody (UCP1, 1:1,000, Sigma Aldrich) over night. Secondary antibody-conjugated with horseradish peroxidase (Santa Cruz Biotechnology) was applied for 1 h at RT and sections were visualized using DAB substrate (Vector Laboratories). Standard haematoxylin and eosin (HE) staining was performed on 5 μM tissue sections.

### Analysis of human adipose tissue

G_q_, *UCP1* and *CIDEA* mRNA expression were measured in abdominal omental and adipose tissue (AT) samples obtained in parallel from 266 donors^45^ who underwent open abdominal surgery for Roux-en-Y bypass, sleeve gastrectomy, explorative laparotomy or elective cholecystectomy. All participants gave their written informed consent before taking part in the study. All investigations have been approved by the ethics committee of the University of Leipzig (363-10-13122010 and 017-12-230112) and were carried out in accordance with the Declaration of Helsinki. Human G_q_, *UCP1* and *CIDEA* mRNA expression was measured by qPCR using Assay-on-Demand gene expression kit (G_q_: forward: 5′-GTGGAGAAGGTGTCTGCTTTTGA-3′; reverse: 5′-ATCTTGTTGCGTAGGCAGGTAGG-3′; UCP1: Hs00222453_m1; CIDEA: Hs00154455_m1; Applied Biosystems, Darmstadt, Germany), and fluorescence was detected on an ABI PRISM 7000 Sequence Detector (Applied Biosystems). G_q_, UCP1 and CIDEA mRNA expression was calculated relative to the mRNA expression of HPRT1 mRNA (Hs01003267_m1; Applied Biosystems).

### Statistics

For cell culture experiments, ‘n' indicates the number of cultures grown and differentiated independently. For experiments with mice, ‘*n*' indicates number of mice per each group.

Single comparisons were analysed using two-tailed student's *t*-test. For RhoA activity assay, one-tailed student's *t*-test was used. Multiple comparisons were analysed using analysis of variance (ANOVA) with Newman-Keuls *post-hoc* test. Values below 0.05 were considered significant. Analyses were performed using GraphPad Prism 5 software. All data are represented as mean±s.e.m. The sample size was chosen based on our previous *in vitro* and *in vivo* studies^39^.

## Additional information

**How to cite this article:** Klepac, K. *et al.* The G_q_ signalling pathway inhibits brown and beige adipose tissue. *Nat. Commun.* 7:10895 doi: 10.1038/ncomms10895 (2016).

## Supplementary Material

Supplementary Information Supplementary Figures 1-4 and Supplementary Tables 1-3

## Figures and Tables

**Table 1:** Classification of GPCR families.

**Receptor class**	**Preadipocytes**	**Brown adipocytes**
Class A	139 (76%)	188 (82%)
Class B	5 (3%)	7 (3%)
Class C	5 (3%)	6 (3%)
Adhesion	20 (11%)	15 (7%)
Frizzled	10 (5%)	11 (5%)
Other	3 (2%)	3 (1%)

GPCR, G protein-coupled receptor.

## References

[b1] Pfeifer A. & Hoffmann L. S. Brown, beige, and white: the new color code of fat and its pharmacological implications. Annu. Rev. Pharmacol. Toxicol. 55, 207–227 (2015). 25149919 10.1146/annurev-pharmtox-010814-124346

[b2] Rosen E. D. & Spiegelman B. M. What we talk about when we talk about fat. Cell 156, 20–44 (2014). 24439368 10.1016/j.cell.2013.12.012 PMC3934003

[b3] Harms M. & Seale P. Brown and beige fat: development, function and therapeutic potential. Nat. Med. 19, 1252–1263 (2013). 24100998 10.1038/nm.3361

[b4] Cannon B. & Nedergaard J. Brown adipose tissue: function and physiological significance. Physiol. Rev. 84, 277–359 (2004). 14715917 10.1152/physrev.00015.2003

[b5] Kajimura S. & Saito M. A new era in brown adipose tissue biology: molecular control of brown fat development and energy homeostasis. Annu. Rev. Physiol. 76, 225–249 (2013). 24188710 10.1146/annurev-physiol-021113-170252 PMC4090362

[b6] Virtanen K. A. *et al.* Functional brown adipose tissue in healthy adults. N. Engl. J. Med. 360, 1518–1525 (2009). 19357407 10.1056/NEJMoa0808949

[b7] van Marken Lichtenbelt W. D. *et al.* Cold-activated brown adipose tissue in healthy men. N. Engl. J. Med. 360, 1500–1508 (2009). 19357405 10.1056/NEJMoa0808718

[b8] Galic S., Oakhill J. S. & Steinberg G. R. Adipose tissue as an endocrine organ. Mol. Cell. Endocrinol. 316, 129–139 (2010). 19723556 10.1016/j.mce.2009.08.018

[b9] Cinti S. Transdifferentiation properties of adipocytes in the adipose organ. Am. J. Physiol. Endocrinol. Metab. 297, E977–E986 (2009). 19458063 10.1152/ajpendo.00183.2009

[b10] Frontini A. & Cinti S. Distribution and development of brown adipocytes in the murine and human adipose organ. Cell. Metab. 11, 253–256 (2010). 20374956 10.1016/j.cmet.2010.03.004

[b11] Kobilka B. K. Structural insights into adrenergic receptor function and pharmacology. Trends. Pharmacol. Sci. 32, 213–218 (2011). 21414670 10.1016/j.tips.2011.02.005 PMC3090711

[b12] Lefkowitz R. J. Seven transmembrane receptors: something old, something new. Acta Physiol. (Oxf.) 190, 9–19 (2007). 17428228 10.1111/j.1365-201X.2007.01693.x

[b13] Latek D., Modzelewska A., Trzaskowski B., Palczewski K. & Filipek S. G protein-coupled receptors--recent advances. Acta. Biochim. Pol. 59, 515–529 (2012). 23251911 PMC4322417

[b14] Wettschureck N. & Offermanns S. Mammalian G proteins and their cell type specific functions. Physiol. Rev. 85, 1159–1204 (2005). 16183910 10.1152/physrev.00003.2005

[b15] Neves S. R., Ram P. T. & Iyengar R. G protein pathways. Science 296, 1636–1639 (2002). 12040175 10.1126/science.1071550

[b16] Buhl A. M., Johnson N. L., Dhanasekaran N. & Johnson G. L. G alpha 12 and G alpha 13 stimulate Rho-dependent stress fiber formation and focal adhesion assembly. J. Biol. Chem. 270, 24631–24634 (1995). 7559569 10.1074/jbc.270.42.24631

[b17] Wang Y., Falting J. M., Mattsson C. L., Holmstrom T. E. & Nedergaard J. In brown adipocytes, adrenergically induced beta(1)-/beta(3)-(Gs)-, alpha(2)-(Gi)- and alpha(1)-(Gq)-signalling to Erk1/2 activation is not mediated via EGF receptor transactivation. Exp. Cell Res. 319, 2718–2727 (2013). 23948306 10.1016/j.yexcr.2013.08.007

[b18] Gnad T. *et al.* Adenosine activates brown adipose tissue and recruits beige adipocytes via A2A receptors. Nature 516, 395–399 (2014). 25317558 10.1038/nature13816

[b19] Sharman J. L. *et al.* IUPHAR-DB: updated database content and new features. Nucleic Acids Res. 41, D1083–D1088 (2013). 23087376 10.1093/nar/gks960 PMC3531077

[b20] Schrage R. *et al.* The experimental power of FR900359 to study Gq-regulated biological processes. Nat. Commun. 6, 10156 (2015). 26658454 10.1038/ncomms10156 PMC4682109

[b21] Conklin B. R. *et al.* Engineering GPCR signalling pathways with RASSLs. Nat. Methods 5, 673–678 (2008). 18668035 10.1038/nmeth.1232 PMC2703467

[b22] Armbruster B. N., Li X., Pausch M. H., Herlitze S. & Roth B. L. Evolving the lock to fit the key to create a family of G protein-coupled receptors potently activated by an inert ligand. Proc. Natl Acad. Sci. USA 104, 5163–5168 (2007). 17360345 10.1073/pnas.0700293104 PMC1829280

[b23] D'Orleans-Juste P., Plante M., Honore J. C., Carrier E. & Labonte J. Synthesis and degradation of endothelin-1. Can. J. Physiol. Pharmacol. 81, 503–510 (2003). 12839262 10.1139/y03-032

[b24] Uchida Y. *et al.* Endothelin-1, but not endothelin-3, suppresses lipoprotein lipase gene expression in brown adipocytes differentiated in culture. Eur. J. Pharmacol. 291, 33–41 (1995). 8549645 10.1016/0922-4106(95)90186-8

[b25] Mitchell J. R. *et al.* Regulation of expression of the lipoprotein lipase gene in brown adipose tissue. Am. J. Physiol. 263, E500–E506 (1992). 1415530 10.1152/ajpendo.1992.263.3.E500

[b26] Haas B. *et al.* Protein kinase g controls brown fat cell differentiation and mitochondrial biogenesis. Sci. Signal. 2, ra78 (2009). 19952371 10.1126/scisignal.2000511

[b27] McDonald M. E. *et al.* Myocardin-related transcription factor A regulates conversion of progenitors to beige adipocytes. Cell 160, 105–118 (2015). 25579684 10.1016/j.cell.2014.12.005 PMC4384505

[b28] Shimizu I. *et al.* Vascular rarefaction mediates whitening of brown fat in obesity. J. Clin. Invest. 124, 2099–2112 (2014). 24713652 10.1172/JCI71643 PMC4001539

[b29] Amisten S. *et al.* An atlas of G-protein coupled receptor expression and function in human subcutaneous adipose tissue. Pharmacol. Ther. 146, 61–93 (2015). 25242198 10.1016/j.pharmthera.2014.09.007

[b30] Regard J. B., Sato I. T. & Coughlin S. R. Anatomical profiling of G protein-coupled receptor expression. Cell 135, 561–571 (2008). 18984166 10.1016/j.cell.2008.08.040 PMC2590943

[b31] Xue R. *et al.* Clonal analyses and gene profiling identify genetic biomarkers of the thermogenic potential of human brown and white preadipocytes. Nat. Med. 21, 760–768 (2015). 26076036 10.1038/nm.3881 PMC4496292

[b32] Pernow J., Shemyakin A. & Bohm F. New perspectives on endothelin-1 in atherosclerosis and diabetes mellitus. Life Sci. 91, 507–516 (2012). 22483688 10.1016/j.lfs.2012.03.029

[b33] Crissey J. M. *et al.* Adipose tissue and vascular phenotypic modulation by voluntary physical activity and dietary restriction in obese insulin-resistant OLETF rats. Am. J. Physiol. Regul. Integr. Comp. Physiol. 306, R596–R606 (2014). 24523340 10.1152/ajpregu.00493.2013 PMC4043131

[b34] Lee Y. H., Petkova A. P., Mottillo E. P. & Granneman J. G. *In vivo* identification of bipotential adipocyte progenitors recruited by beta3-adrenoceptor activation and high-fat feeding. Cell Metab. 15, 480–491 (2012). 22482730 10.1016/j.cmet.2012.03.009 PMC3322390

[b35] Li Y. Q. *et al.* Gq/11alpha and Gsalpha mediate distinct physiological responses to central melanocortins. J. Clin. Invest. 126, 40–49 (2015). 26595811 10.1172/JCI76348 PMC4701544

[b36] Jennissen K. *et al.* A VASP-Rac-soluble guanylyl cyclase pathway controls cGMP production in adipocytes. Sci. Signal. 5, ra62 (2012). 22932701 10.1126/scisignal.2002867

[b37] Chen Y. *et al.* miR-155 regulates differentiation of brown and beige adipocytes via a bistable circuit. Nat. Commun. 4, 1769 (2013). 23612310 10.1038/ncomms2742 PMC3644088

[b38] Elabd C. *et al.* Human multipotent adipose-derived stem cells differentiate into functional brown adipocytes. Stem Cells 27, 2753–2760 (2009). 19697348 10.1002/stem.200

[b39] Hoffmann L. S. *et al.* Stimulation of soluble guanylyl cyclase protects against obesity by recruiting brown adipose tissue. Nat. Commun. 6, 7235 (2015). 26011238 10.1038/ncomms8235 PMC4455111

[b40] Betz M. J. *et al.* Presence of brown adipocytes in retroperitoneal fat from patients with benign adrenal tumors: relationship with outdoor temperature. J. Clin. Endocrinol. Metab. 98, 4097–4104 (2013). 23744406 10.1210/jc.2012-3535

[b41] Insel P. A. *et al.* G protein-coupled receptor (GPCR) expression in native cells: "novel" endoGPCRs as physiologic regulators and therapeutic targets. Mol. Pharmacol. 88, 181–187 (2015). 25737495 10.1124/mol.115.098129 PMC4468643

[b42] Kalinec G., Nazarali A. J., Hermouet S., Xu N. & Gutkind J. S. Mutated alpha subunit of the Gq protein induces malignant transformation in NIH 3T3 cells. Mol. Cell. Biol. 12, 4687–4693 (1992). 1328859 10.1128/mcb.12.10.4687-4693.1992 PMC360395

[b43] Schroder R. *et al.* Deconvolution of complex G protein-coupled receptor signalling in live cells using dynamic mass redistribution measurements. Nat. Biotechnol. 28, 943–949 (2010). 20711173 10.1038/nbt.1671

[b44] Pfeifer A. Lentiviral transgenesis. Transgenic. Res. 13, 513–522 (2004). 15672832 10.1007/s11248-004-2735-5

[b45] Kloting N. *et al.* Insulin-sensitive obesity. Am. J. Physiol. Endocrinol. Metab. 299, E506–E515 (2010). 20570822 10.1152/ajpendo.00586.2009

