# Supplementary material for: The Gq signalling pathway inhibits brown and beige adipose tissue
Source: Nat Commun. 2016 Mar 9;7:10895. doi: 10.1038/ncomms10895 (PMC4786868; doi:10.1038/ncomms10895)
Supplement: Supplementary Information — Supplementary Figures 1-4 and Supplementary Tables 1-3 [file ncomms10895-s1.pdf]

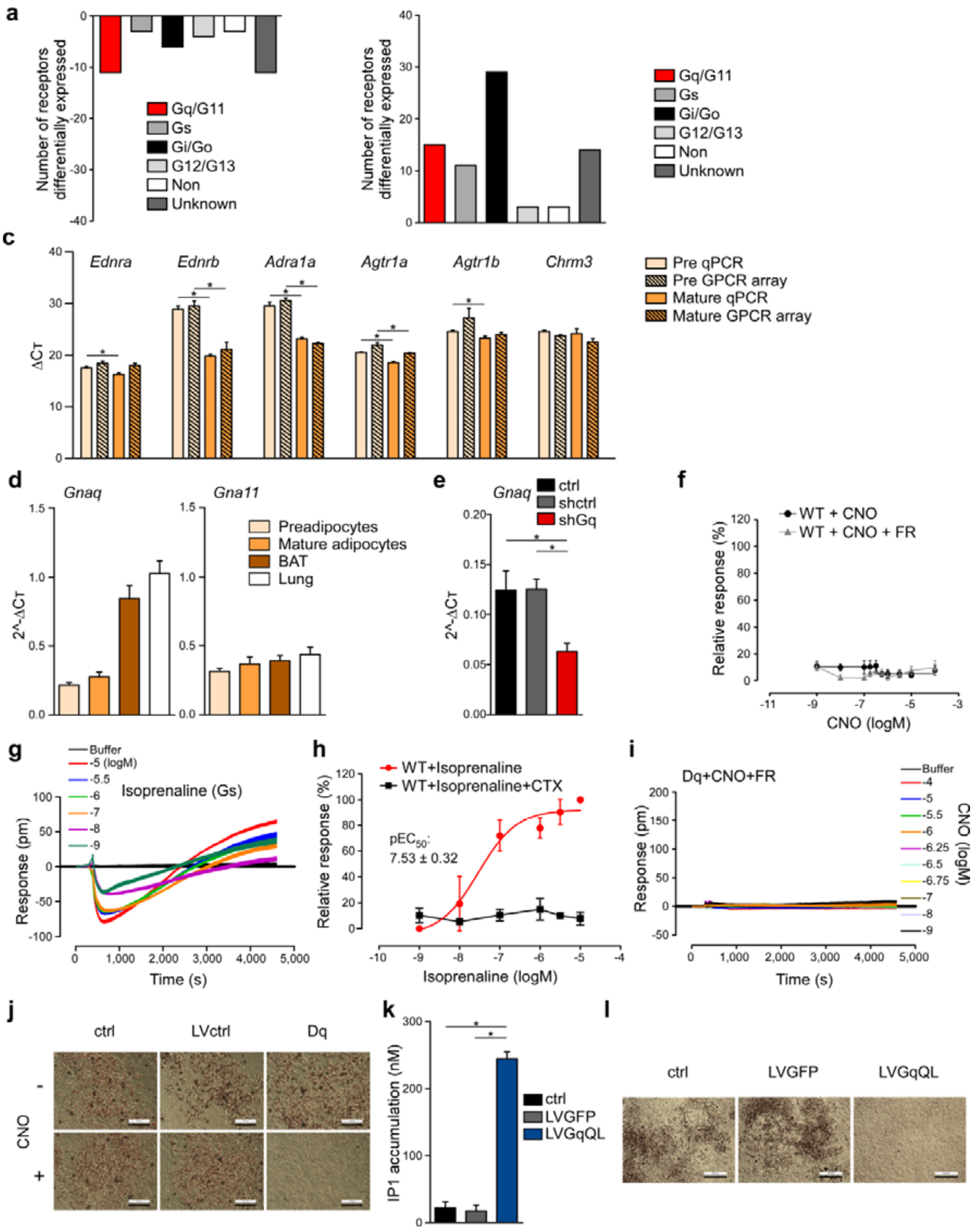

Supplementary Figure 1: GPCR profiling and G_q signaling in murine brown adipocytes (BA). a, Number of GPCRs with 2-fold lower expression in mature BA vs. preadipocytes. **b,** Number of GPCRs with 2-fold higher expression in mature BA vs. preadipocytes. **c,** GPCR validation; the mRNA expression of G_q coupled GPCRS detected in murine preadipoctes (pre) and mature brown adipocytes (mature) with qPCR or with a GPCR array. Ct values were normalized to *18S*. t-test, *P<0.05. **d,**

Relative mRNA expression of G_q and G_{11} in preadipocytes, BA and BAT. ANOVA, *P<0.05. **e,** Relative mRNA expression of G_q in untransduced cells and cells transduced with shctrl or shGq. ANOVA, *P<0.05. **f,** Concentration-effect curve for wild-type cells treated with CNO, in presence and absence of FR. **g,** Representative traces of label-free DMR in brown preadipocytes stimulated with isoprenaline. **h,** Concentration-effect curve of WT cells stimulated with isoprenaline, in presence and absence of G_s inhibitor cholera toxin (CTX). **i,** Representative traces of cells expressing Dq stimulated with CNO and FR. **j,** Oil Red O stain of BA transduced with control virus (LVctrl) or virus containing Dq and differentiated in presence or absence of CNO. Scale bar, 10 µm. **k,** IP1 accumulation (nM) in untransduced preadipocytes and preadipocytes stabily expressing GFP (LVGFP) or GqQL (LVGqQL). ANOVA, *P<0.05. **l,** Oil Red O stain of BA transduced with LVGFP or LVGqQL. Scale bar, 20 µm. **a-b,** n=3 independent arrays. **c,d,** n = 4. **e,** n = 5. **f-i,** n = 3. **j,** n = 4. **k,** n = 3. **l** = 5. All data are shown as mean±s.e.m.

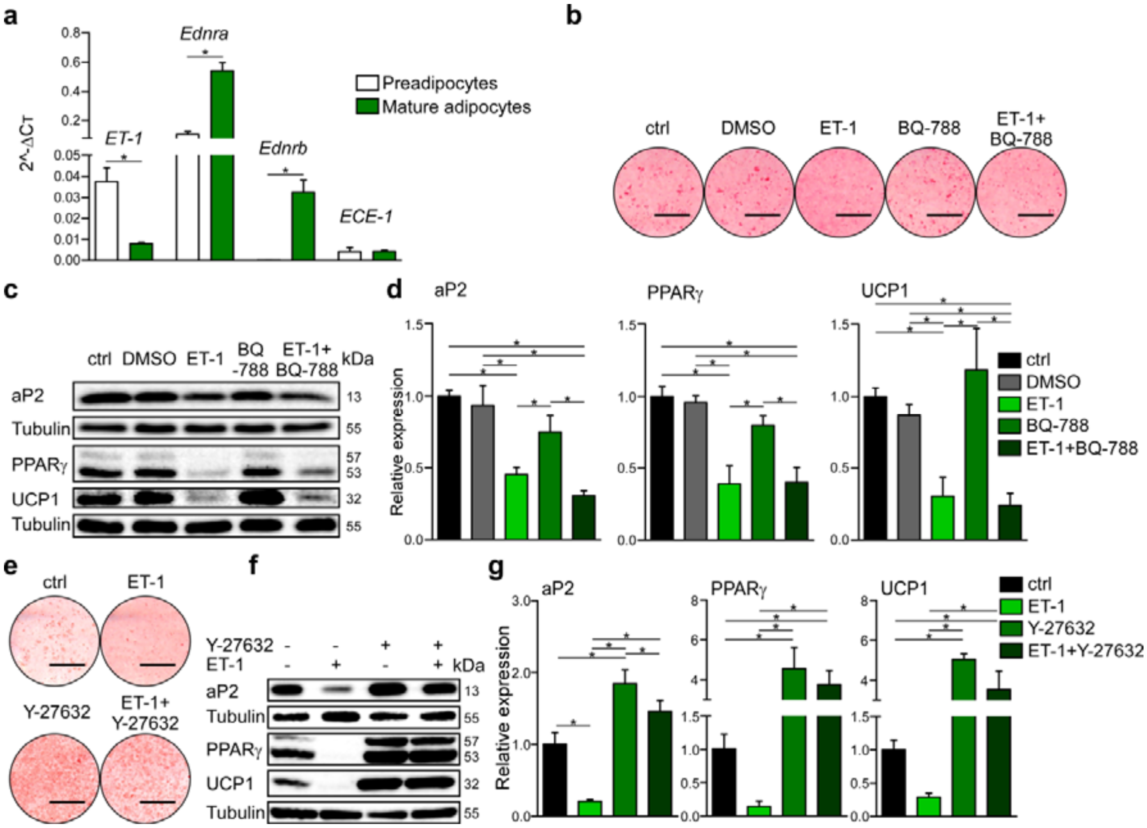

Supplementary Figure 2: Role of Endothelin-1 in differentiation and downstream signaling of G_q. a, Relative mRNA expression of endothelin system in preadipocytes and mature BA: *endothelin-1 (ET-1)*, *endothelin receptor type A and B (Ednra, Ednrb)*, *Endothelin converting enzyme-1 (ECE-1)*. t-test, *P<0.05. **b,** Oil Red O stain of BA chronically treated with ET-1, Ednrb inhibitor BQ-788, or both. Scale bar, 1 cm. **c,d,** Representative immunoblots (**c**) and quantification (**d**) of adipogenic markers and UCP1 after treatment of BA with and without ET-1, BQ-788 or both. ANOVA, *P<0.05. **e,** Oil Red O stain of BA chronically treated with ET-1, Y-27632, or both. Scale bar, 1 cm. **f,g,** Representative immunoblots (**f**) and quantification (**g**) of adipogenic markers (aP2; PPARγ) and UCP1 after treatment of BA with and without ET-1, Y-27632 or both. ANOVA, *P<0.05. **a,** n = 4. **b-g,** n = 3. All data are shown as mean±s.e.m.

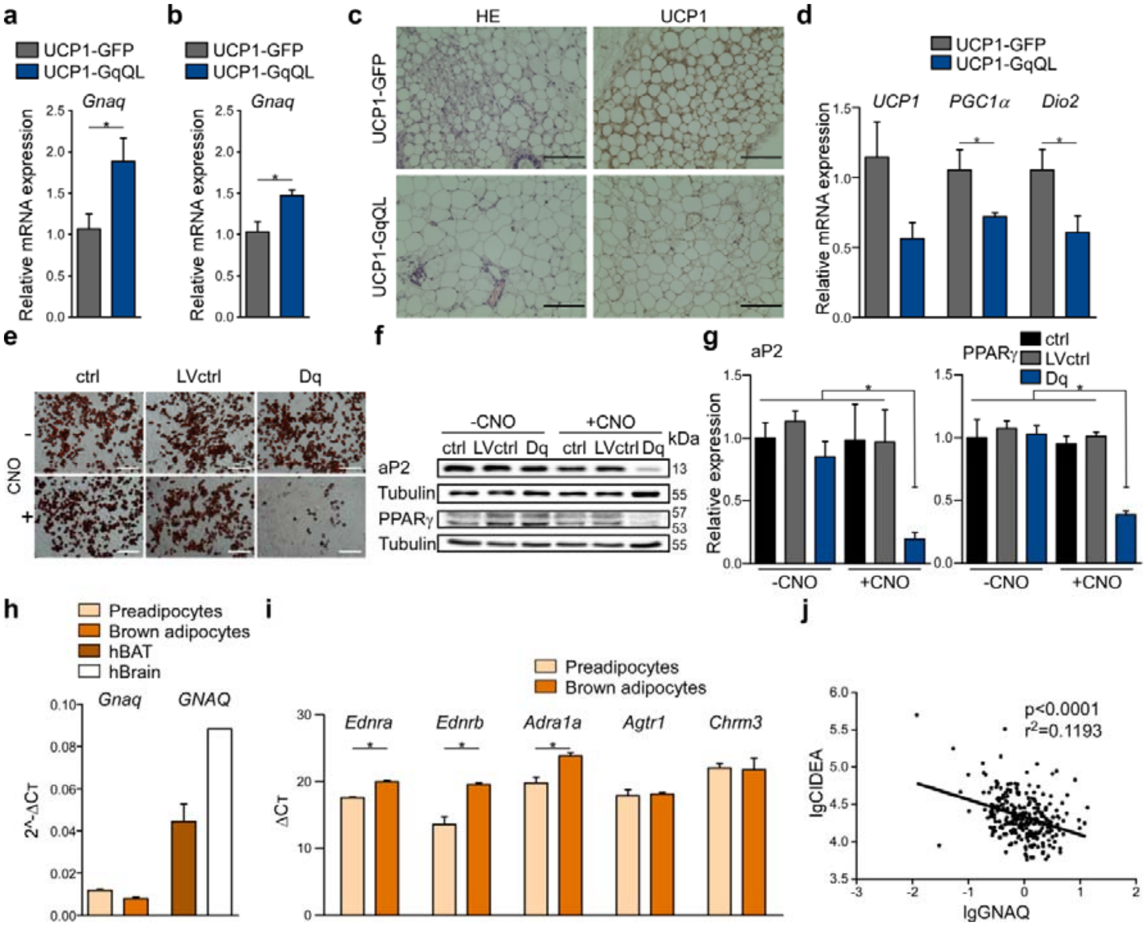

Supplementary Figure 3: **G_q activation reduces browning of WAT *in vivo.* a,b,** Relative mRNA expression of G_q in inguinal (**a**) and gonadal/visceral (**b**) WAT of UCP1-GFP and UCP1-GqQL mice. t-test, **P*<0.05. **c,** Representative HE and UCP1 staining of gonadal/visceral WAT in UCP1-GFP and UCP1-GqQL mice after cold stimulation. Scale bar, 100 µm. **d,** Relative mRNA expression of thermogenic markers in gonadal/visceral WAT of UCP1-GqQL relative to UCP1-GFP mice. t-test, **P*<0.05. **e,** Oil Red O stain of WAi transduced with control virus (LVctrl) or virus containing Dq differentiated in presence or absence of CNO. Scale bar, 20 µm. **f,g,** Representative immunoblots (**f**) and quantification (**g**) of aP2 and PPARγ in WAi transduced with LVctrl or Dq and differentiated in presence or absence of CNO. ANOVA, **P*<0.05. **h,** Relative mRNA expression of G_q in human preadipocytes, mature adipocytes and human BAT normalized to *ACTB (β-actin)*. **i,** mRNA expression of G_q coupled GPCRs in preadipocyte hMADS and mature hMADS determined by qPCR. Ct values were normalized to *28S*. t-test, **P*<0.05. **j**, Correlation of G_q and *CIDEA* expression in human WAT. **a-d,** 6 animals per group were analysed. **e-i**, n=4-5. **j,** 266 human abdominal WAT samples were analyzed. All data are shown as mean±s.e.m.

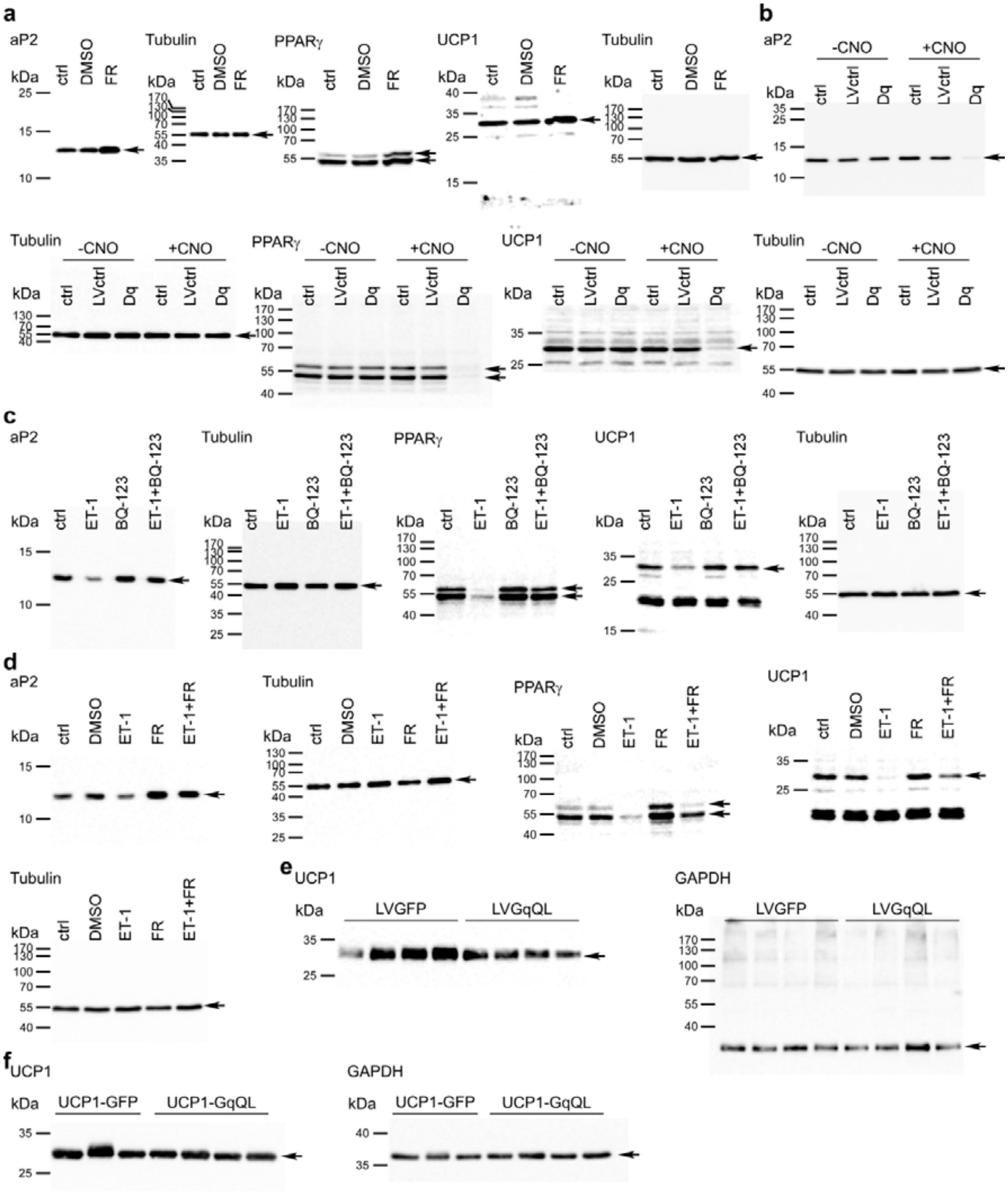

Supplementary Figure 4: Complete immunoblots of sections shown in Figures 1-3. a, Complete immunoblots of aP2, PPARγ, UCP1 and Tubulin of sections shown in **Fig. 1e**. **b,** Complete immunoblots of aP2, PPARγ, UCP1 and Tubulin of sections shown in **Fig. 1k**. **c,** Complete immunoblots of aP2, PPARγ, UCP1 and Tubulin of sections shown in **Fig. 2c**. **d,** Complete immunoblots of aP2, PPARγ, UCP1 and Tubulin of sections shown in **Fig. 2f**. **e,** Representative immunoblots of UCP1 and GAPDH of sections shown in **Fig. 3d**. **f,** Representative immunoblots of UCP1 and GAPDH of sections shown in **Fig. 3g**.

Supplementary Table 1: GPCRs expressed in brown preadipocytes

Name	ΔC_t	Primary Linkage or role
ADCYAP1R1	21.7	Gs
ADMR	21.8	Unknown
ADORA2A	22.6	Gs
ADORA2B	19.8	Gs
ADRA1B	19.2	Gq/11
ADRA1D	16.9	Gq/11
ADRA2A	21.5	Gi/0
ADRA2B	22.7	Gi/0
ADRB1	19.8	Gs
ADRB2	22.9	Gs
ADRB3	26.2	Gs
AGTR1A	21.9	Gi/0, Gq/11
AGTR2	20.7	Gi/0
AGTRL1	24.7	Gi/0
AVPR1A	20.0	Gq/11
BAI1 (ADGRB1)	24.4	Unknown
BAI2 (ADGRB2)	23.4	G16 (Gq)
BDKRB1	23.3	Gi/0, Gq/11
BDKRB2	24.2	Gs, Gi/0, Gq/11
C3AR1	21.9	Unknown
C5R1	21.2	Gi/0
CALCRL	19.0	Gs
CCR1	24.7	Gi/0
CCR2	22.4	Gi/0
CCR3	24.4	Gi/0
CCR4	24.9	Gi/0
CCR8	23.4	Gi/0
CCR9	22.9	Gi/0
CCRL1 (CX3CR1)	22.7	Gi/0
CCRL2	22.5	Unknown
CD97 (ADGRE5)	16.1	G12/13
CELSR1	22.6	Other
CELSR2	22.2	Other
CHRM1	25.5	Gq/11
CHRM2	22.7	Gi/0
CHRM3	23.8	Gq/11

CHRM4	22.2	Gi/0
CHRM5	24.4	Gq/11
CMKLR1	21.5	Gi/0
CMKOR1 (ACKR3)	24.1	Unknown
CNR1	22.4	Gi/0
CX3CR1	22.5	Gi/0
CXCR3	26.4	Gi/0
CXCR6	22.1	Gi/0
DRD4	25.1	Gi/0
DUSP2	26.3	Unknown
EDG1 (S1PR1)	19.6	Gi/0
EDG2 (LPAR1)	17.2	Gi/0, Gq/11, G12/13
EDG3 (S1PR3)	22.3	Gi/0, Gq/11, G12/13
EDG4 (LPAR2)	18.7	Gi/0, Gq/11, G12/13
EDG5 (S1PR2)	24.4	Gq/11, G12/13, Gs
EDG6 (S1PR4)	24.5	Gi/0, G12/13
EDG7 (LPAR3)	21.7	Gi/0, Gq/11
EDG8 (S1PR5)	24.0	Gi/0, G12/13
EDNRA	18.4	Gq/11
ELTD1	22.9	Unknown
EMR1	24.8	Unknown
F2R (PAR1)	14.8	Gq/11,Gi/0,G12/13
F2RL1 (PAR2)	19.5	Gq/11,Gi/0
FPR1	22.8	Gi/0
FPR-RS2 (FPR3)	23.9	Gi/0
FPR-RS3	23.9	Unknown
FPR-RS6	25.5	Unknown
FPR-RS7	25.0	Unknown
FZD1	16.6	WNT
FZD2	17.1	WNT
FZD3	18.5	WNT, Gs
FZD4	16.6	G12/13, Non-canonical signaling, WNT
FZD5	18.7	WNT
FZD6	17.7	Gi/0, Gq/11, WNT
FZD7	16.9	Gs, Gi/0, WNT
FZD8	18.0	WNT
FZD9	21.6	Unknown
GABBR1	17.5	Gi/0
GALR2	23.3	Gq/11
GPBAR1	20.7	Gs

GPR1	23.3	Unknown
GPR108	17.5	Unknown
GPR114 (ADGRG5)	23.3	Unknown
GPR115 (ADGRF4)	24.0	Unknown
GPR116 (ADGRF5)	23.1	Unknown
GPR119	26.3	Gs
GPR124 (ADGRA2)	15.6	Unknown
GPR125 (ADGRA3)	16.0	Unknown
GPR133 (ADGRD1)	22.8	Gs
GPR135	20.3	Unknown
GPR137	17.7	Unknown
GPR137b	16.0	Unknown
GPR146	21.3	Unknown
GPR149	24.2	Unknown
GPR151	21.0	Unknown
GPR152	25.0	Unknown
GPR153	18.0	Unknown
GPR156	22.5	Unknown
GPR160	23.1	Unknown
GPR161	17.8	Unknown
GPR162	23.0	Unknown
GPR17	23.4	Gi/0
GPR173	20.7	Unknown
GPR175 (TPRA1)	19.8	Unknown
GPR176	16.3	Unknown
GPR18	21.5	Gi/0, Gq/11
GPR183 (EBI2)	20.4	Gi/0
GPR19	26.6	Unknown
GPR20	23.6	Gi/0
GPR21	20.1	Gq/11
GPR23 (LPAR4)	17.7	Gi/0, Gq/11, Gs, G12/13
GPR3	23.2	Gs
GPR30 (GPER)	21.3	Gi/0
GPR34	21.5	Gi/0
GPR35	24.3	Unknown
GPR39	20.2	Gq/11
GPR4	21.1	Gs, Gq/11, Gi/0, G12/13
GPR44 (PTGDR2)	24.1	Gi/0
GPR56 (ADGRG1)	19.9	Gq/11, G12/13
GPR61	21.4	Constitutive?

GPR62	20.4	Unknown
GPR63	20.3	Unknown
GPR64 (ADGRG2)	22.8	Gs, Gq
GPR65	23.4	Gs
GPR75	19.8	Gq/11
GPR81 (HCAR1)	22.8	Gi/0
GPR82	19.0	Unknown
GPR84	24.2	Gi/0
GPR85	18.4	Unknown
GPR92 (LPAR5)	24.4	Gq/11, G12/13
GPRC5A	17.9	Unknown
GPRC5B	17.4	Unknown
GRM6	20.0	Gi/0
HRH1	21.5	Gq/11
HRH2	26.7	Gq/11
HTR1B	21.1	Gi/0
HTR1D	23.8	Gi/0
HTR2A	18.8	Gq/11
KISS1R	25.4	Gq/11
LENG4	16.8	Unknown
LGR4	20.8	Unknown
LGR5	21.4	Unknown
LGR6	22.7	Unknown
LGR7 (RXFP1)	23.6	Gs, Gi/0
LPHN1 (ADGRL1)	16.0	Gq/11
LPHN2 (ADGRL2)	15.6	Unknown
LPHN3 (ADGRL3)	20.3	Unknown
LTB4R2	23.7	Gi/0
MAS1	21.3	Gi/0, Gq/11
MASS1 (ADGRV1)	22.1	Unknown
MC1R	22.9	Gs
MC3R	24.5	Gs
MC4R	25.7	Gs
MRGPRB5	25.3	Unknown
MRGPRE	23.2	Unknown
MRGPRF	20.5	Unknown
MTNR1A	24.3	Gi/0
NPBWR1	24.7	Gi/0
NPY1R	24.4	Gi/0
NPY6R	23.2	Gi/0

OPRL1	25.9	Gi/0
P2RY14	25.2	Gi/0
P2RY2	23.7	Gq/11
P2RY5 (LPA6)	15.9	Gs, Gi/0, G12/13
P2RY6	23.1	Gq/11
PRLHR	25.7	Gq/11
PROKR1	20.1	Gq/11
PROKR2	24.4	Gq/11
PTAFR	26.4	Gq/11, Gi/0, independent
PTGER1	22.1	Gq/11
PTGER4	17.7	Gs
PTGIR	25.8	Gs
PTHR1	19.6	Gs
PTHR2	23.9	Gs, Gq/11
RXFP3	21.5	Gi/0
RXFP4	22.6	Gi/0
SMO	15.0	Gi/0, G12/13
SSTR3	24.4	Gi/0
SSTR4	22.9	Gi/0
TACR2	23.4	Gs, Gq/11
TACR3	25.0	Gq/11
TBXA2R	20.1	Gq/11
TM7SF3	16.3	Unknown
VIPR1	23.0	Gs
VIPR2	22.4	Gs
XCR1	25.3	Gi/0
ACTB	8.5	Reference
GAPDH	8.8	Reference
HPRT	14.2	Reference

Supplementary Table 2: GPCRs expressed in mature brown adipocytes

**Name**	**ΔC_t**	**Primary Linkage or role**
ADCYAP1R1	18.3	Gs
ADMR	22.6	Unknown
ADORA1	21.3	Gi/0
ADORA2A	19.6	Gs
ADORA2B	20.7	Gs
ADRA1A	22.3	Gq/11
ADRA1B	19.6	Gq/11
ADRA1D	18.7	Gq/11
ADRA2A	21.4	Gi/0
ADRA2B	20.8	Gi/0
ADRA2C	21.5	Gi/0
ADRB1	19.8	Gs
ADRB2	20.6	Gs
ADRB3	20.2	Gs
AGTR1A	20.4	Gi/0, Gq/11
AGTR1B	24.0	Gi/0, Gq/11
AGTR2	14.4	Gi/0
AGTRL1	21.7	Gi/0
AVPR1A	17.8	Gq/11
BAI2 (ADGRB2)	22.7	G16 (Gq)
BDKRB1	22.2	Gi/0, Gq/11
BDKRB2	22.2	Gs, Gi/0, Gq/11
C3AR1	18.9	Unknown
C5R1	16.9	Gi/0
CALCRL	19.0	Gs
CCR1	22.3	Gi/0
CCR2	21.6	Gi/0
CCR3	21.2	Gi/0
CCR4	22.7	Gi/0
CCR5	22.6	Gi/0
CCR6	24.7	Gi/0
CCR8	22.1	Gi/0
CCR9	21.7	Gi/0
CCRL1 (CX3CR1)	22.2	Gi/0
CCRL2	19.8	Unknown
CD97 (ADGRE5)	16.8	G12/13
CELSR2	20.4	Other

CHRM1	22.3	Gq/11
CHRM2	22.7	Gi/0
CHRM3	21.6	Gq/11
CHRM4	21.2	Gi/0
CHRM5	22.8	Gq/11
CMKLR1	20.6	Gi/0
CMKOR1 (ACKR3)	24.3	Unknown
CNR1	22.3	Gi/0
CNR2	23.5	Gi/0
CX3CR1	22.2	Gi/0
CXCR3	21.4	Gi/0
CXCR6	20.8	Gi/0
CYSLTR1	22.0	Gq/11
CYSLTR2	23.7	Gq/11
DARC	24.4	Unknown
DRD1	24.1	Gs
DRD5	24.8	Gs
EDG1 (S1PR1)	19.9	Gi/0
EDG2 (LPAR1)	17.2	Gi/0, Gq/11, G12/13
EDG3 (S1PR3)	22.4	Gi/0, Gq/11, G12/13
EDG4 (LPAR2)	20.8	Gi/0, Gq/11, G12/13
EDG5 (S1PR2)	24.9	Gq/11, G12/13, Gs
EDG6 (S1PR4)	21.2	Gi/0, G12/13
EDG7 (LPAR3)	23.6	Gi/0, Gq/11
EDG8 (S1PR5)	22.5	Gi/0, G12/13
EDNRA	18.0	Gq/11
EDNRB	21.1	Gq/11, Gi/0, Gs
EMR1	18.0	Unknown
F2R (PAR1)	17.6	Gq/11,Gi/0,G12/13
F2RL1 (PAR2)	22.2	Gq/11,Gi/0
FFAR1	24.5	Gq/11
FFAR2	22.8	Gq/11
FPR1	22.0	Gi/0
FPRL1 (FPR2)	23.0	Gi/0
FPR-RS2 (FPR3)	22.1	Gi/0
FPR-RS3	22.2	Unknown
FPR-RS4	23.0	Unknown
FPR-RS6	22.6	Unknown
FPR-RS7	23.5	Unknown
FZD1	16.7	WNT

FZD10	20.4	Beta-catenin
FZD2	18.2	WNT
FZD3	20.0	WNT, Gs
FZD4	16.0	G12/13, Non-canonical signaling, WNT
FZD5	18.4	WNT
FZD6	21.1	Gi/0, Gq/11, WNT
FZD7	16.7	Gs, Gi/0, WNT
FZD8	18.5	WNT
FZD9	21.3	Unknown
GABBR1	18.4	Gi/0
GALR2	21.9	Gq/11
GIPR	25.0	Gs
GNRHR	26.6	Gq/11
GPBAR1	20.3	Gs
GPR1	23.8	Unknown
GPR108	16.9	Unknown
GPR109a (HCAR2)	21.6	Gi/0
GPR115 (ADGRF4)	27.3	Unknown
GPR116 (ADGRF5)	24.4	Unknown
GPR119	22.1	Gs
GPR12	23.9	Gs, Gi/0
GPR120 (FFAR4)	17.8	Gq/11
GPR124 (ADGRA2)	16.2	Unknown
GPR125 (ADGRA3)	17.9	Unknown
GPR132	23.8	Gs, G12/13
GPR133 (ADGRD1)	19.4	Gs
GPR135	20.8	Unknown
GPR137	18.4	Unknown
GPR137b	16.1	Unknown
GPR141	22.2	Unknown
GPR146	19.3	Unknown
GPR150	23.4	Unknown
GPR151	20.8	Unknown
GPR152	22.6	Unknown
GPR153	17.7	Unknown
GPR156	23.5	Unknown
GPR160	22.0	Unknown
GPR161	19.5	Unknown
GPR162	22.0	Unknown
GPR17	22.7	Gi/0

GPR171	23.2	Unknown
GPR173	21.1	Unknown
GPR175 (TPRA1)	20.9	Unknown
GPR176	19.3	Unknown
GPR18	20.3	Gi/0, Gq/11
GPR183 (EBI2)	19.5	Gi/0
GPR19	22.7	Unknown
GPR20	22.9	Gi/0
GPR21	20.2	Gq/11
GPR23 (LPAR4)	18.0	Gi/0, Gq/11, Gs, G12/13
GPR3	22.8	Gs
GPR30 (GPER)	21.8	Gi/0
GPR33	24.4	Gi/0
GPR34	20.9	Gi/0
GPR35	22.4	Unknown
GPR39	22.0	Gq/11
GPR4	20.4	Gs, Gq/11, Gi/0, G12/13
GPR44 (PTGDR2)	22.8	Gi/0
GPR45	22.8	Unknown
GPR55	24.1	Gq/11, G12/13
GPR56 (ADGRG1)	23.7	Gq/11, G12/13
GPR6	22.3	Gs, Gi/0
GPR61	19.6	Constitutive?
GPR62	20.0	Unknown
GPR63	20.0	Unknown
GPR64 (ADGRG2)	23.8	Gs, Gq
GPR65	19.7	Gs
GPR75	19.9	Gq/11
GPR77 (C5AR2)	23.1	Arrestin?
GPR81 (HCAR1)	19.4	Gi/0
GPR82	18.8	Unknown
GPR84	23.2	Gi/0
GPR85	19.6	Unknown
GPR87	22.0	Unknown
GPR92 (LPAR5)	20.6	Gq/11, G12/13
GPRC5A	18.6	Unknown
GPRC5B	17.7	Unknown
GPRC5C	23.5	Unknown
GRM3	26.3	Gi/0
HRH1	21.1	Gq/11

HRH2	21.5	Gq/11
HTR1A	23.3	Gi/0
HTR1B	21.5	Gi/0
HTR1D	22.7	Gi/0
HTR1F	25.0	Gi/0
HTR2A	20.2	Gq/11
HTR2B	26.5	Gq/11
IL8RA	22.4	Gi/0
IL8RB	21.0	Gi/0
KISS1R	22.5	Gq/11
LENG4	17.5	Unknown
LGR4	18.2	Unknown
LGR6	19.2	Unknown
LPHN1 (ADGRL1)	17.0	Gq/11
LPHN2 (ADGRL2)	16.8	Unknown
LPHN3 (ADGRL3)	21.3	Unknown
LTB4R	22.3	Gq/11, Gi/0
LTB4R2	21.5	Gi/0
MAS1	21.0	Gi/0, Gq/11
MASS1 (ADGRV1)	25.1	Unknown
MC1R	21.9	Gs
MC2R	19.9	Gs
MC3R	24.3	Gs
MC4R	21.6	Gs
MC5R	20.9	Gs
MRGPRA2	22.1	Unknown
MRGPRA4	24.1	Unknown
MRGPRA6	23.0	Unknown
MRGPRB1	24.3	Unknown
MRGPRB2	24.9	Gq/11
MRGPRB4	24.1	Unknown
MRGPRB5	23.1	Unknown
MRGPRD	21.5	Gi/0, Gq/11
MRGPRE	22.3	Unknown
MRGPRF	19.9	Unknown
MRGPRG	23.5	Unknown
NPY1R	24.2	Gi/0
NPY2R	24.5	Gi/0
NPY6R	23.0	Gi/0
OXGR1	*25.5*	Gq/11

OXTR	24.2	Gq/11
P2RY13	22.8	Gi/0
P2RY14	22.8	Gi/0
P2RY2	22.7	Gq/11
P2RY5 (LPA6)	16.7	Gs, Gi/0, G12/13
P2RY6	20.0	Gq/11
PPYR1	21.2	Gi/0
PRLHR	23.2	Gq/11
PROKR1	19.8	Gq/11
PTAFR	20.2	Gq/11, Gi/0, independent
PTGER1	22.7	Gq/11
PTGER2	23.1	Gs
PTGER3	21.5	Gi/0
PTGER4	18.6	Gs
PTGFR	21.5	Gq/11
PTHR1	17.3	Gs
RXFP3	21.6	Gi/0
RXFP4	21.8	Gi/0
SCTR	22.1	Gs
SMO	16.7	Gi/0, G12/13
SSTR1	20.5	Gi/0
SSTR3	22.6	Gi/0
SSTR4	21.3	Gi/0
SSTR5	22.3	Gi/0
SUCNR1	24.7	Gi/0, Gq/11
TAAR1	22.2	Gs
TAAR9	22.7	Unknown
TBXA2R	21.4	Gq/11
TM7SF3	16.6	Unknown
TSHR	18.8	Gs
VIPR1	25.2	Gs
VIPR2	21.0	Gs
XCR1	22.2	Gi/0
ACTB	9.8	Reference
GAPDH	9.7	Reference
HPRT	15.0	Reference

Supplementary Table 3: qPCR primer sequences

Name	Species	Forward	Reverse
28S	human	GCC TAG CAG CCG ACT TAG AA	AAA TCA CAT CGC GTC AAC AC
ADRA1A	human	ACT TCT CAG TGA GGC TCC T	AGG GCT TGA AAT CAG GGA AG
AGTR1	human	CCG CCC CTC AGA TAA TGT AAG	TCG AAC ATG TCA CTC AAC CTC
aP2	human	TGT GCA GAA ATG GGA TGG AAA	CAA CGT CCC TTG GCT TAT GCT
CHRM3	human	CAC CAA GTT ACC CTC ATC GG	AAA ACT GCC TCC ATC GTC C
EDNRA	human	TGC CCT CAG TGA ACA TCT TAA G	CAT CGG TTC TTG TCC ATC TCG
EDNRB	human	GTC CCA ATA TCT TGA TCG CCA G	AAG GCA CCA GCT TAC ACA TCT
GAPDH	human	TGG TCT CCT CTG ACT TCA AC	GTG AGG GTC TCT CTC TTC CT
Gq	human	GTG GAG AAG GTG TCT GCT TTT GA	ATC TTG TTG CGT AGG CAG GTA GG
PPARg	human	AGC CTC ATG AAG AGC CTT CCA	TCC GGA AGA AAC CCT TGC A
UCP1	human	TGC CCA ACT GTG CAA TGA A	CCA GGA TCC AAG TCGCAA GA
18S	murine	CCA TCC AAT CGG TAGT AGC G	GTA ACC CGT TGA ACC CCA TT
ADRA1A	murine	TTC CTC GTG ATG CCC ATT G	GCT TTC TTG AAC TCC TGG CTG
AGTR1A	murine	GAC CAA CTC AAC CCA GAA AAG C	ATC ACC ACC AAG CTG TTT CC
AGTR1B	murine	TGG CTT GGC TAG TTT GCC G	ACC CAG TCC AAT GGG GAG T
CHRM3	murine	ACA GTA GCA GTG ACA GTT GG	GAG TTG AGG ATG GTG CTA TGA C
DIO2	murine	GCG ATG GCA AAG ATA GGT GA	GAA TGG AGC TGG GTG TAG CA
ECE-1	murine	ATG ACG CCG CCC ATG GTG AAC	TGG TTG GGC TAA GAC ATA AC
EDNRA	murine	GCT GGT TCC CTC TTC ACT TAA GC	TCA TGG TTG CCA GGT TAA TGC
EDNRB	murine	TGT GCT CTA AGT ATT AGA CAG ATA TCG A	GGC TGT CTT GTA AAA CTG CAT GA
ET1	murine	GGA AAC TAC GAA GGT TGG AGG C	CTG TAG AAG CCA CAC AGA TGG TCT
G11	murine	CAC AGG GGC CTC TTC CAA AT	CAG GCA AGG TGCA GTG ACT A
Gq	murine	GTG CCT GCA CAC CTT ACT CT	AAC TAA GAA GCC CTT CCC GC
HPRT	murine	GTC CCA GCG TCG TGA TTA GC	TCA TGA CAT CTC GAG CAA GTC TTT
HPRT	murine	ACA TTG TGG CCC TCT GTG TGC TCA	CTG GCA ACA TCA ACA GGA CTC CTC GT
mutGq	murine	TAG CCG ACC CTT CCT ATC TGC	TCT GAC CTT TGG CCC CCT A
ND5	murine	AGC ATT CGG AAG CAT CCT TG	TTG TGA GGA CTG GAA TGC TG
NUFA	murine	AGA CGC ATC TCT GGT GTC AA	GCC AGG AAA ATG CTT CCT TA
PGC1a	murine	GCACACACCGCAATTCTCCCTTGTA	ACG CTG TCC CAT GAG GTA TTG ACC A
UCP1	murine	TAA GCC GGC TGA GAT CTT GT	GGC CTC TAC GAC TCA GTC CA
